# Dirhodium C–H Functionalization of Hole-Transport Materials

**DOI:** 10.1021/acs.joc.2c02888

**Published:** 2023-03-15

**Authors:** Farzaneh Saeedifard, Yasir Naeem, Yannick T. Boni, Yi-Chien Chang, Junxiang Zhang, Yadong Zhang, Bernard Kippelen, Stephen Barlow, Huw M. L. Davies, Seth R. Marder

**Affiliations:** † School of Chemistry and Biochemistry, Georgia Institute of Technology, Atlanta, Georgia 30332, United States; ‡ Renewable and Sustainable Energy Institute, University of Colorado Boulder, Boulder, Colorado 80303, United States; § Department of Chemistry, Emory University, Atlanta, Georgia 30322, United States; ∥ School of Electrical and Computer Engineering, Center for Organic Photonics and Electronics (COPE), Georgia Institute of Technology, Atlanta, Georgia 30332, United States; ⊥ Department of Chemical and Biological Engineering, University of Colorado Boulder, Boulder, Colorado 80303, United States; # Materials Science and Engineering Program, University of Colorado Boulder, Boulder, Colorado 80303, United States; ∇ Department of Chemistry, University of Colorado Boulder, Boulder, Colorado 80303, United States

## Abstract

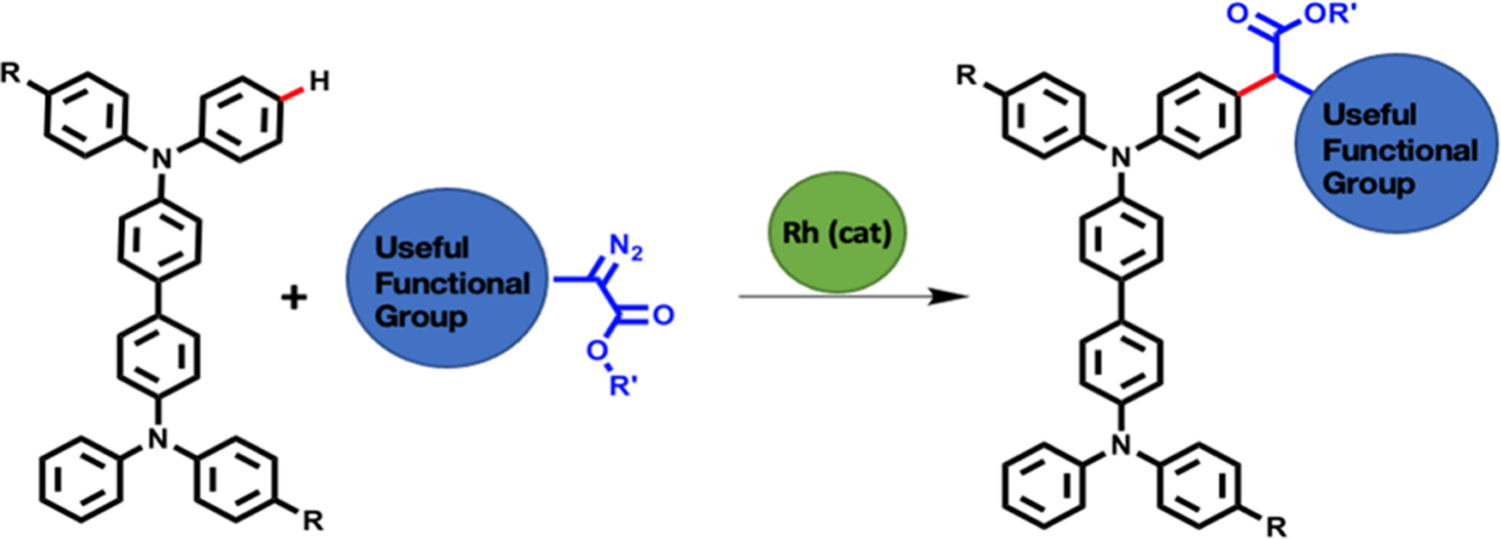

Hole-transport materials (HTMs) based on triarylamine derivatives play important roles in organic electronics applications including organic light-emitting diodes and perovskite solar cells. For some applications, triarylamine derivatives bearing appropriate binding groups have been used to functionalize surfaces, while others have been incorporated as side chains into polymers to manipulate the processibility of HTMs for device applications. However, only a few approaches have been used to incorporate a single surface-binding group or polymerizable group into triarylamine materials. Here, we report that Rh-carbenoid chemistry can be used to insert carboxylic esters and norbornene functional groups into sp^2^ C–H bonds of a simple triarylamine and a 4,4′-bis(diarylamino)biphenyl, respectively. The norbenene-functionalized monomer was polymerized by ring-opening metathesis; the electrochemical, optical, and charge-transport properties of these materials were similar to those of related materials synthesized by conventional means. This method potentially offers straightforward access to a diverse range of HTMs with different functional groups.

## Introduction

**Chart 1:**
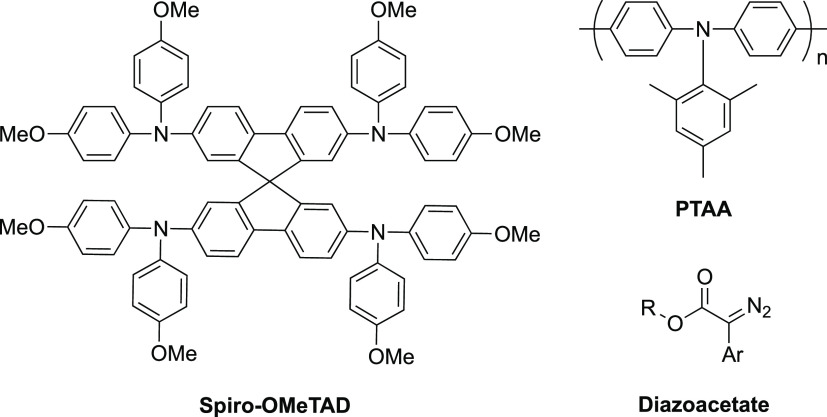
Examples of Hole-Transporting Triarylamine Derivatives and a Generic Diazoacetate Precursor to a Reactive Carbene

Hole-transport materials (HTMs) based on triarylamines, often bis(diarylamino)biphenyls in particular, have been used in a variety of organic electronic devices, particularly organic light-emitting diodes,^1^ and, more recently, perovskite solar cells (PSCs).^2,3^ These materials include small molecules such as spiro-OMeTAD, and main-chain polymers such as PTAA (Chart 1). A third approach uses pendant triarylamine-based moieties, linked by side chains to a non-redox-active main chain; this approach is attractive in that the HT moiety and polymer main chain can be varied to tune redox and morphological properties and comonomers bearing crosslinking groups can be introduced to allow for thermal or photochemical insolubilization subsequent to deposition, which can be valuable in fabricating multilayer devices from solution.^4,5^ In addition, monolayers consisting of HTM moieties bound to an ITO surface have recently been found to be useful in PSCs; for these monolayers, both phosphonic acid and carboxylic acid binding groups have been used.^6,7^

Attaching a single polymerizable group or a surface-anchoring functional group to a simple triarylamine or to a bis(diarylamino)biphenyl often requires multistep synthesis. For example, polymerizable bis(diarylamino)biphenyl derivatives have been obtained through the incorporation of a protected alcohol on one of the bromoarenes used in multiple successive Buchwald–Hartwig Pd-catalyzed C–N bond forming reactions, prior to deprotection and esterification with methacrylic acid.^8^ On the other hand, a more easily synthesized more symmetric bis(diarylamino)biphenyl moiety can be converted via a Vilsmeier reaction to a formyl derivative, which can be subsequently reduced to an alcohol, which can then be esterified.^5^

Recently, the C–H insertion chemistry of rhodium carbenes has emerged as a powerful synthetic method that offers new strategies for the construction of complex targets.^9−12^ Diazo compounds (Chart 1) are among the most frequently used precursors for transition-metal-catalyzed carbene reactions; on reaction with some transition-metal complexes, notably dirhodium tetracarboxylate catalysts,^13^ they are transformed to highly electrophilic metal carbenes, which can undergo a variety of useful transformations, including C–H insertion.^10^ Significant progress has been made in the development of both C(sp^3^)-H and C(sp^2^)-H insertion reactions. For example, asymmetric arylation of diazo compounds with aniline derivatives, catalyzed by an achiral dirhodium complex and a chiral spiro phosphoric acid has been reported.^14^ For these types of reactions, a delicate balance of electronic and steric effects controls the chemoselectivity displayed by these carbenoids. C–H insertion is preferred at sites that stabilize positive charge buildup on the carbon.

In this article, we report the application of dirhodium carbene chemistry for the insertion of important carboxylic esters and norbornene functional groups into the C(sp^2^)–H bonds of a simple triarylamine and a bis(diarylamino)biphenyl moiety, respectively, as a simple means of synthesizing HTMs. This method is likely to be particularly advantageous when one wishes to attach the same functional group to a range of different triarylamine derivatives.

## Results and Discussion

### Catalyst Screening

**Scheme 1:**
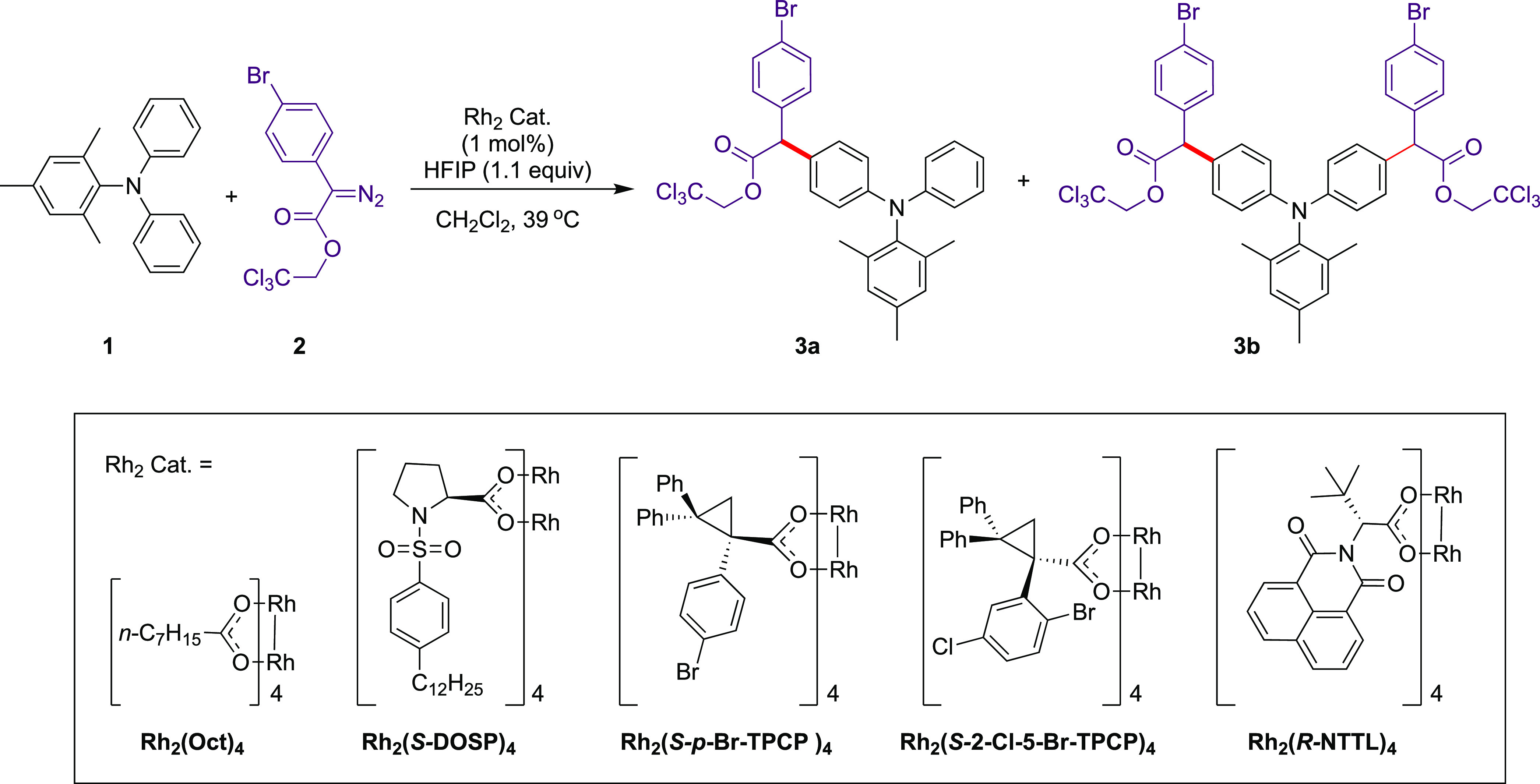
Catalyst Screening for Triarylamine sp^2^ C–H Functionalization

It has been demonstrated that electron-rich aromatic rings are susceptible to electrophilic substitution reactions with donor/acceptor-substituted carbenoids using dirhodium catalysts if the aromatic ring is not sterically encumbered.^15^ Therefore, we have used this chemistry to test the feasibility of the insertion of carboxylic esters into C–H bonds of triarylamine derivatives. **2** (Scheme 1) was chosen as the desired diazo substrate because the ester functional group can easily be hydrolyzed to a carboxylic acid (which could, in principle, be used as a surface-binding group). The reaction of a simple triarylamine, **1**, with 1.5 equiv of **2** in the presence of an achiral catalyst Rh_2_(Oct)_4_ (rhodium(II) octanoate) (1 mol %) and HFIP (1,1,1,3,3,3-hexafluoro-2-propanol) resulted in dirhodium-catalyzed C–H insertion into para-C(sp^2^)-H bond of **1** to give a mixture of single-insertion product (**3a**) in 42% yield and the double-insertion product (**3b**) in 20% yield (Table 1, entry 1). The role of HFIP is to avoid interference by traces of water contaminant, and the diazo compound was added slowly to avoid carbene dimer formation.^16^ We examined a range of chiral dirhodium catalysts to test the enantioselectivity of the reactions. As summarized in Table 1 (entries 2–5), zero to moderate enantiomeric excesses were observed. Although Rh_2_(*S*-DOSP)_4_ and Rh_2_(*R*-NTTL)_4_ gave no enantioselectivity, they both afforded relatively high yields of **3a**, and thus Rh_2_(*S*-DOSP)_4_ was used for the reactions described in the following sections.

**Table 1:** Catalyst Screening According to Scheme 1^a^

entry	Rh_2_ cat.	**3a** % yield^b^	**3a** % ee^c^	**3b** % yield^b^
1	Rh_2_(Oct)_4_	42	0	20
2	Rh_2_(*S*-DOSP)_4_	65	0	31
3	Rh_2_(*S*-*p*-Br-TPCP)_4_	48	20	19
4	Rh_2_(*S*-2-Cl-5-Br-TPCP)_4_	44	0	33
5	Rh_2_(*R*-NTTL)_4_	68	4	30

a Reaction conditions: A solution of **2** (0.15 mmol) in dry CH_2_Cl_2_ (2.50 mL) was added via an automatic syringe pump over 3 h to a mixture of **1** (0.10 mmol) and Rh_2_ cat. (1 mol %) in dry CH_2_Cl_2_ (0.50 mL) along with HFIP (1,1,1,3,3,3-hexafluoro-2-propanol, 1.10 equiv) at 39 °C. The mixture was stirred for an additional 1 h at 39 °C under Ar.

b Isolated yields.

c Enantiomeric excess determined by chiral HPLC.

### Triarylamine Scope

**Scheme 2:**
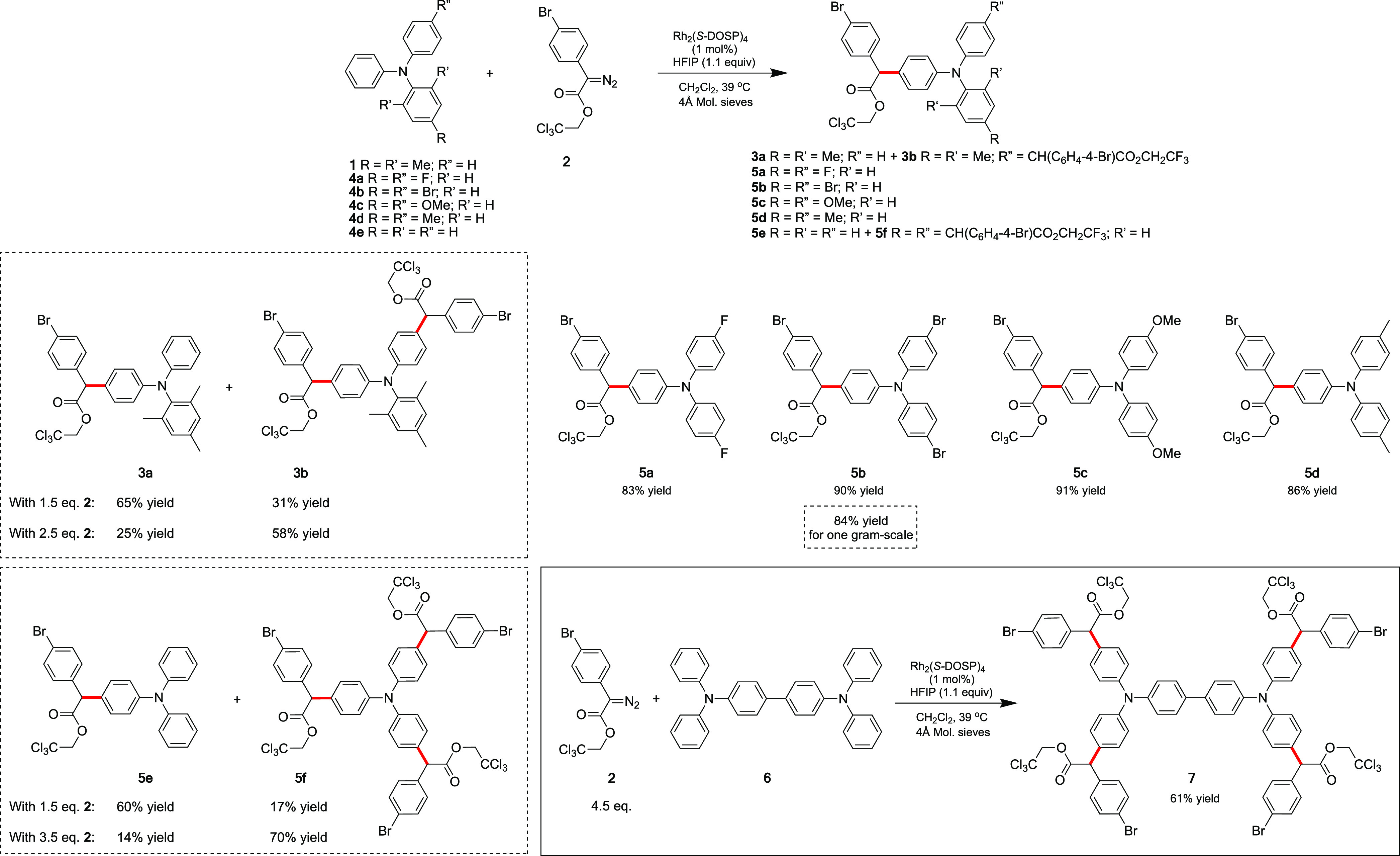
sp^2^ C–H Functionalization of a Variety of Triarylamines

Having established the optimal conditions to selectively introduce donor/acceptor carbene fragments derived from **2** onto triarylamine **1**, we explored the scope of the Rh_2_(*S*-DOSP)-catalyzed reaction with different triarylamines (**1**, **4a–e**, Scheme 2), which produced the corresponding C–H-functionalized products (**3a**, **3b**, **5a–f**) in excellent yields. As noted above and in Table 1, the 2,4,6-substituted triarylamine **1** provided **3a** with an isolated yield of 65% as a major product and **3b** with an isolated yield of 31% as a minor product. An excess amount of diazo compound **2** provided **3a** in an isolated yield of 25% as a minor product and **3b** with an isolated yield of 58% as a major product. 4,4′-Disubstituted triarylamines **4a–d**, which only have one unblocked *para* position, afforded singly functionalized products, all in isolated yields >80% regardless of the electron-donating or -withdrawing characteristics of the 4,4′-substituents. Triphenylamine **4e**, containing potentially three reactive *para* positions, reacted with 1.5 equiv **2** to give **5e** in an isolated yield of 60% as a major product and triply substituted **5f** in an isolated yield of 17% as a minor product. Excess diazo compound provides **5f** with an isolated yield of 70% as a major product and **5e** with an isolated yield of 14% as a minor product. We also extended the reaction to a 4,4′-bis(diarylamino)biphenyl substrate **6** in which there are potentially four reactive *para* positions; the tetra-functionalized product **7** was obtained in an isolated yield of 61%.

### Monomer and Polymer Synthesis and Characterization

**Scheme 3:**
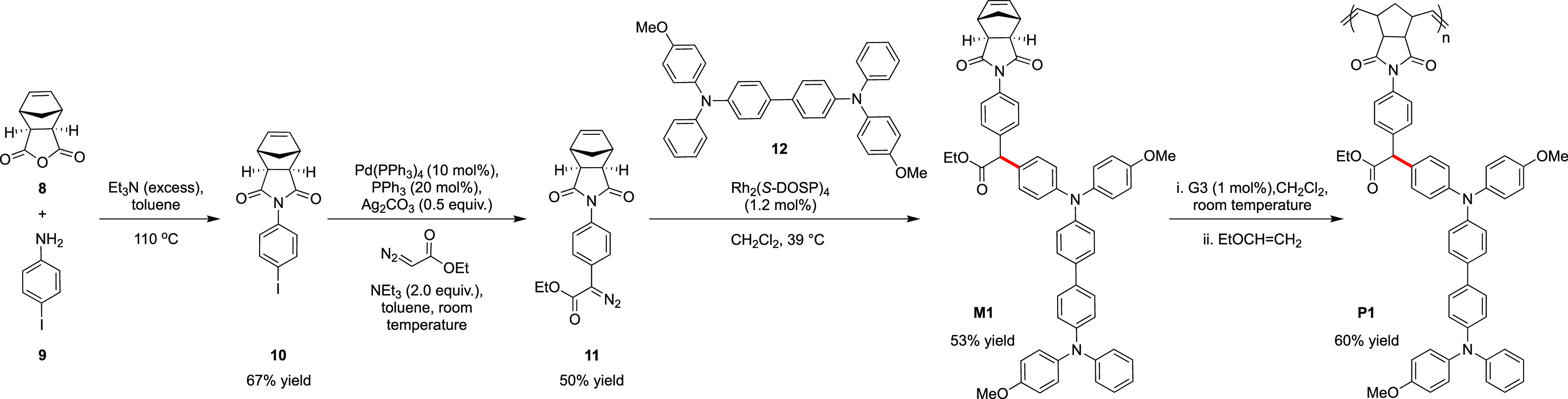
sp^2^ C–H Functionalization of a Bis(Diarylamino)biphenyl with a Norbornene

Since bis(diarylamino)biphenyl side-chain polymers have been applied in both OLEDs and PSCs, we next tested the feasibility of using the dirhodium chemistry to insert a polymerizable norbornene group into the C–H bond of a bis(diarylamino)biphenyl derivative using Rh_2_(*S*-DOSP)_4_. Palladium-catalyzed C–H functionalization has been used for the synthesis of ethyl aryldiazoacetates from aryliodides and ethyl diazoacetate.^17^ Therefore, an aryl diazoacetate containing the norbornene functional group should be obtainable from an iodoaryl norbornene derivative with ethyl diazoacetate. *cis*-5-Norbornene-*exo*-2,3-dicarboxylic anhydride was chosen as a source of the polymerizable group since it is commercially available and exhibits rapid reactivity toward ring-opening metathesis polymerization (ROMP).^18^ Condensation of the anhydride **8** with 4-iodoaniline **9** generated the desired iodoaryl norbornene derivative **10** with 67% yield (Scheme 3). This intermediate underwent the reaction with ethyl diazoacetate in the presence of Pd(PPh_3_)_4_ and Ag_2_CO_3_ to produce **11** with 50% yield.

Slow addition of **11** to the solution of MeO-TPD, **12**, in CH_2_Cl_2_ at 40 °C led to the formation of two compounds, as indicated by the observation of two TLC spots, one corresponding to single insertion into MeO-TPD starting material **12** (desired product), and one showing a mass spectrum consistent with the formation of the double-insertion product. After purification, the desired single-insertion product **M1** was obtained (Scheme 3). Although synthesis of the diazo precursor requires several steps, this route is potentially advantageous if one wants to derivatize multiple different bis(diarylamino)biphenyls with the same functional group in that the derivatization is achieved in a single step, whereas via the formylation and reduction route mentioned above, several steps must be performed on each bis(diarylamino)biphenyl.

**Figure 1:**
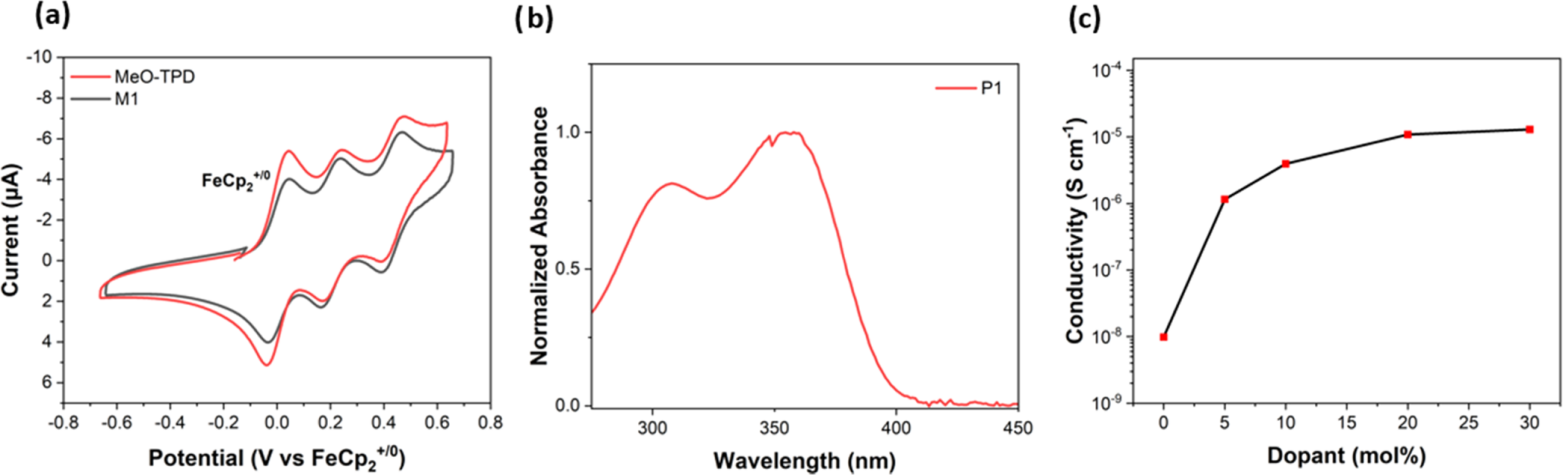
(a) Cyclic voltammetry of **M1** and MeO-TPD (**12**) in dichloromethane (10^–3^ M) with 0.1 M Bu_4_NPF_6_ and ferrocene as a reference, (b) UV–vis spectra of **P1** in chlorobenzene, and (c) electrical conductivity of Mo(tfd-COCF_3_)_3_-doped **P1**.

Electrochemical measurements on the monomer **M1** in CH_2_Cl_2_/0.1 M Bu_4_NPF_6_ indicate two successive reversible oxidations at *E* = +0.21 and +0.42 V vs internal FeCp_2_^+/0^, which is the same as we obtain for the MeO-TPD starting material measured in the same way, Figure 1a, indicating that the polymerizable group can be attached with negligible change in the electronic properties of the bis(diarylamino)moiety.

**Chart 2:**
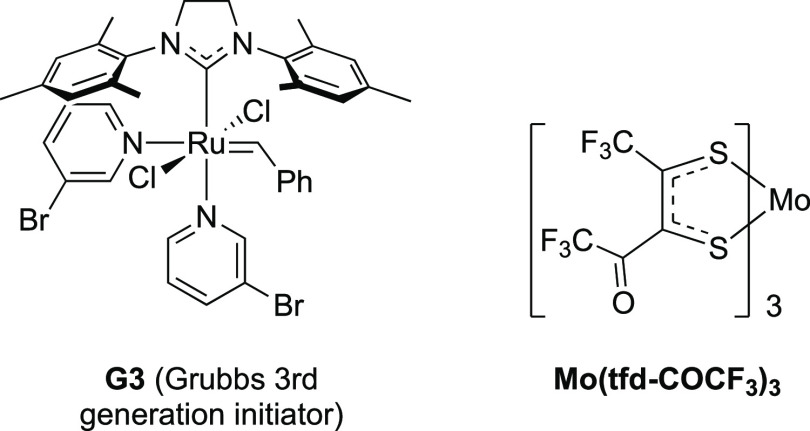
Structure of Initiator Used to Synthesize P1 and p-Dopant Used to Increase Its Conductivity

**P1** was successfully synthesized using the Grubbs third-generation initiator G3 (Scheme 3, Chart 2). The polymer was soluble in a range of organic solvents including THF, chloroform, dichloromethane, and dichlorobenzene. A broadening of the ^1^H NMR peaks was observed, consistent with the formation of a polymer. Moreover, an appreciable downfield chemical shift corresponding to the norbornene alkene protons was observed in the ^1^H NMR spectrum of the polymer, consistent with the ring opening of the norbornene. Gel permeation chromatography (in CHCl_3_) also indicated the formation of a polymer and suggested Mw = 150 kDa and PDI = 1.1. The UV–vis absorption spectrum of **P1** (Figure 1b) is similar to that of other bis(diarylamino)biphenyl side-chain polymers in the literature.^4^

The in-plane bulk conductivities of films of **P1** p-doped with Mo(tfd-COCF_3_)_3_ and spin-coated on glass substrates were measured with a four-point probe as a function of dopant concentration (Figure 1c). Undoped **P1** films yield an in-plane conductivity of 9.8 × 10^–9^ S cm^–1^, consistent with what is expected for nominally undoped films. When Mo(tfd-COCF_3_)_3_ is incorporated into the casting solution (20 mol %), the conductivity of **P1** increases by up to 4 orders of magnitude to a value of 1.1 × 10^–5^ ± 1.1 × 10^–7^ S cm^–1^. The increase in conductivity is clearly consistent with doping. Moreover, the resulting conductivity is of the same order of magnitude as that of Li[N(SO_2_CF_3_)_2_]/air-doped spiro-OMeTAD, which is widely and successfully used as an HTM in PSCs.^19^ However, the best conductivity value is 1 order of magnitude lower than that previously reported for a similar side-chain polymer (the copolymer of a bis(diarylamino)biphenyl-acrylate monomer and a cinnamate-acrylate monomer) doped with 2,3,5,6-tetrafluoro-7,7,8,8-tetracyanoquinodimethane (20 mol %).^4^ The results are not directly comparable in that the dopants are different and the backbones quite different in terms of rigidity, but the dipole moments associated with the polar moieties (−CO_2_Et and −N(CO)_2_−) present in **P1** may lead to the energetic disorder that adversely affects mobility.^20^

## Conclusions

In summary, we have described the use and practicality of C–H functionalization chemistry to functionalize triarylamine derivatives with groups important for materials applications—carboxylic esters and norbornene. Specifically, a variety of triarylamines was found to undergo C(sp^2^)-H activation using a diazoacetate bearing a protected carboxylic acid functional group in the presence of Rh_2_(S-DOSP)_4_ (1 mol %). The reaction of a bis(diarylamino)biphenyl with a new diazoacetate containing a norbornene functional group offered one step to the access to the monomer (**M1**) for the synthesis of hole-transport polymer (**P1**) using ROMP. A comparison of UV–vis and cyclic voltammetry data confirms that this chemistry had little effect on the electronic structure of the HTM moieties. The electrical conductivity of the polymer doped using Mo(tfd-COCF_3_)_3_ is comparable to those exhibited by some HTMs successfully used in PSCs. The C–H functionalization chemistry used in this work could be useful for the insertion of other important functional groups on HTMs, such as phosphonic esters for surface modifications, or benzocyclobutane for thermal crosslinking of HTMs.

## Experimental Section

### General Information

Chemicals were obtained from commercial sources and used as received unless stated otherwise. MeO-TPD (**12**),^21^ compound **2**,^17^ Mo(tfd-COCF_3_)_3_,^22^ Rh_2_(*S*-DOSP)_4_,^23^ Rh_2_(*S*-*p*-Br-TPCP)_4_,^24^ Rh_2_(*S*-2-Cl-5-Br-TPCP)_4_,^25^ and Rh_2_(*R*-NTTL)_4_^26^ were synthesized based on the literature reports. All operations involved in synthesis were performed under an atmosphere of nitrogen or argon using standard Schlenk techniques. ^1^H NMR and ^13^C NMR spectra were recorded in CDCl_3_ on Varian 500, 600, or 700 MHz spectrometers. The chemical shifts (δ) are reported in parts per million (ppm).

Infrared (IR) spectra were collected on a Nicolet Impact Series 10 FT-IR. Mass spectrometric determinations were carried out on a Thermo Fisher Scientific Exactive Plus Orbitrap Mass spectrometer with electrospray ionization (ESI) or atmospheric pressure chemical ionization (APCI), or (for **10**, **11** and, **M1**) a Thermo Fisher Scientific Q-Exactive HF Quadrupole-Orbitrap Mass spectrometer with ESI.

Absorption spectra were measured with a Cary 5000 UV–vis/NIR spectrometer. The samples were dissolved in anhydrous and degassed chlorobenzene. Cyclic voltammetry was performed using a BAS potentiostat with a glassy carbon working electrode, platinum counter electrode, and a Ag wire reference electrode at a scan rate of 100 mV s^–1^. Anhydrous and degassed CH_2_Cl_2_ solutions containing 0.1 M tetrabutylammonium hexafluorophosphate as an electrolyte were used to dissolve **M1** (10^–3^ M). Ferrocene was used as the reference.

For electrical conductivity measurements, **P1** and the dopant were dissolved in chlorobenzene at concentrations of 10 and 15 mg mL^–1^, respectively. Both solutions were stirred overnight in the glovebox. The dopant solution was then added to the **P1** solution for various mol %, and the doped solutions were stirred at 70 °C for 1 h. The solutions were spin-coated at 1000 rpm, 2000 rpm s^–1^, and 20 s inside the glovebox, and the films were dried for 1 h before sheet resistance measurement. The thickness of the films was about 35 nm and measured by a profilometer in air. The resistance was measured by a four-point probe with a Keithley 6430 glovebox.

### General Procedure for C–H Functionalization with 2,2,2-Trichloroethyl Esters

A clean oven-dried and flame-dried 8.0 mL scintillation vial (vial-A) equipped with a few activated 4 Å molecular sieves and a magnetic stir bar was evacuated and purged with argon (2–3 times). After cooling down to room temperature, the relevant triarylamine (**1**, **4a–e**, or **6**, 1.0 equiv, 0.200 mmol) followed by Rh_2_(*S*-DOSP)_4_ (3.79 mg, 0.01 equiv), and 1,1,1,3,3,3-hexafluoro-2-propanol (HFIP) (1.1 equiv) were then added. The vial was once again evacuated and purged with argon (3–5 times) and anhydrous CH_2_Cl_2_ (0.5 mL) was added. The vial and its contents were then set to stir at reflux (39 °C) under an argon atmosphere using a hotplate. To a second oven-dried vial (vial-B) that was evacuated and purged with argon was added 2,2,2-trichloroethyl-2-(4-bromophenyl)-2-diazoacetate, **2** (1.0 to 4.5 equiv). Vial-B and its contents were evacuated and purged with argon (2–3 times), and anhydrous CH_2_Cl_2_ (2.0 mL) was then added to obtain a 0.1 M solution of the diazo compound. The 0.1 M solution was transferred into a 5.0 mL plastic syringe (12.46 mm diameter). Using a well-calibrated syringe pump, slow addition of the diazo solution into the stirring solution of vial-A under an inert atmosphere was initiated. After the complete addition of the solution (3 h), the residual diazo compound in the 5.0 mL plastic syringe was rinsed with anhydrous CH_2_Cl_2_ (0.5 mL) and transferred dropwise into the stirring reaction mixture of vial-A. An additional 30 min was allowed before concentrating the solution under reduced pressure. Purification by flash column chromatography on silica gel (hexane/EtOAc) was used to afford the final product.

### Gram-Scale Procedure for C–H Functionalization with 2,2,2-Trichloroethyl Esters

A clean oven-dried and flame-dried 100.0 mL round-bottom flask (flask-A) equipped with activated 4 Å molecular sieves and a magnetic stir bar was evacuated and purged with argon (2–3 times). After cooling down to room temperature, **4b** (1.04 g, 2.48 mmol) followed by Rh_2_(*S*-DOSP)_4_ (47.0 mg, 0.01 equiv), and 1,1,1,3,3,3-hexafluoro-2-propanol (HFIP) (1.1 equiv) were then added. The flask was once again evacuated and purged with argon (3–5 times) and anhydrous CH_2_Cl_2_ (10.0 mL) was added. The flask and its contents were then set to stir at reflux (39 °C) under an argon atmosphere using a hotplate. To a second oven-dried round-bottom flask (flask-B) that was evacuated and purged with argon was added 2,2,2-trichloroethyl-2-(4-bromophenyl)-2-diazoacetate, **2** (0.924 g, 1.0 equiv, 2.48 mmol). Flask-B and its contents were evacuated and purged with argon (2–3 times), and anhydrous CH_2_Cl_2_ (25.0 mL) was then added to obtain a solution of the diazo compound. The solution was transferred into a 30.0 mL plastic syringe. Using a well-calibrated syringe pump, slow addition of the diazo solution into the stirring solution of flask-A under an inert atmosphere was initiated. After the complete addition of the solution (3 h), the residual diazo compound in the 30.0 mL plastic syringe was rinsed with anhydrous CH_2_Cl_2_ (1.0 mL) and transferred dropwise into the stirring reaction mixture of flask-A. An additional 30 min was allowed before concentrating the solution under reduced pressure. Purification by flash column chromatography on silica gel (hexanes: EtOAc) was used to afford the final product.

#### 2,2,2-Trichloroethyl-2-(4-bromophenyl)-2-(4-(mesityl(phenyl)amino)phenyl)acetate (**3a**) and bis(2,2,2-Trichloroethyl)-2,2′-((4-mesitylazanediyl)bis(4,1-phenylene))bis(2-(4-bromophenyl)acetate) (**3b**)

The material was purified by flash chromatography (SiO_2_; hexanes/EtOAc, 10:0 to 9:1 gradient; *R*_f_ = 0.62 in 9:1 hexanes/EtOAc), which afforded major product **3a** as a colorless gel (82 mg, 65%): ^1^H NMR (600 MHz, CDCl_3_) δ 7.47 (d, *J* = 8.5 Hz, 2H), 7.27–7.24 (m, 2H), 7.18 (t, *J* = 8.0 Hz, 2H), 7.13 (d, *J* = 8.8 Hz, 2H), 6.97 (d, *J* = 8.9 Hz, 2H), 6.94–6.90 (m, 4H), 6.88 (t, *J* = 7.3 Hz, 1H), 5.02 (s, 1H), 4.79 (s, 2H), 2.32 (s, 3H), 1.97 (s, 6H). ^13^C{^1^H} NMR (101 MHz, CDCl_3_) δ 171.1, 146.0, 140.2, 137.9, 137.4, 137.3, 132.1, 130.7, 130.3, 129.6, 129.4, 129.2, 121.9, 121.3, 120.1, 119.7, 95.0, 74.7, 55.9, 21.4, 18.9. HRMS (ESI) calcd for C_31_H_27_BrCl_3_NO_2_ [M + H]^+^ 630.0363, found, 630.0385. IR (neat): 2950, 1752, 1593, 1506, 1490, 1406, 1374, 1374, 1307, 1260, 1178, 1128, 1074, 1028, 1011, 908, 853, 828, 755, 732, 695, 605, 671, 551, 497 cm^–1^. HPLC analysis: (R,R-Whelk column, 1% isopropanol in hexane, 1.0 mL min^–1^, 1 mg mL^–1^, 15 min, UV 230 nm) retention times of 5.8 min (major) and 6.5 min (minor) 20% ee when using Rh_2_(S-p-Br-TPCP)_4_ in place of Rh_2_(S-DOSP)_4_. The minor product **3b** (*R*_f_ = 0.39 in 9:1 hexanes/EtOAc) was obtained from the same column as **3a** as a colorless gel (61 mg, 31%): ^1^H NMR (600 MHz, CDCl_3_) δ 7.46 (d, *J* = 8.5 Hz, 4H), 7.25 (d, *J* = 8.5 Hz, 5H), 7.12 (d, *J* = 8.6 Hz, 4H), 6.91 (d, *J* = 8.9 Hz, 6H), 5.02 (s, 2H), 4.78 (s, 4H), 2.31 (s, 3H), 1.94 (s, 6H). ^13^C{^1^H} NMR (151 MHz, CDCl_3_) δ 171.0, 145.7, 139.9, 137.8, 137.4, 137.3, 132.1, 130.7, 130.3, 129.6, 129.6, 121.9, 119.9, 95.0, 74.7, 55.9, 21.4, 18.8. HRMS (ESI) calcd for C_41_H_33_Br_2_Cl_6_NO_4_ [M + H]^+^ 970.8907, found, 970.8888. IR (neat): 2951, 1749, 1601, 1504, 1487, 1406, 1369, 1307, 1260, 1179, 1124, 1073, 1026, 1011, 907, 853, 829, 763, 730, 649, 571, 552, 529, 496, 449 cm^–1^. When an excess amount of **2** (2.5 equiv) was used the isolated yield of **3b** was (113 mg, 58%) that of **3a** is (31 mg, 25%).

#### 2,2,2-Trichloroethyl-2-(4-(bis(4-fluorophenyl)amino)phenyl)-2-(4-bromophenyl)acetate (**5a**)

The material was purified by flash chromatography (SiO_2_; hexanes/EtOAc, 10:0 to 9:1 gradient; *R*_f_ = 0.62 in 9:1 hexanes/EtOAc), which afforded **5a** as a colorless foam (104 mg, 83% yield). ^1^H NMR (400 MHz, CDCl_3_) δ 7.47 (d, *J* = 8.5 Hz, 2H), 7.25 (d, *J* = 8.5 Hz, 2H), 7.15 (d, *J* = 8.6 Hz, 2H), 7.04–6.99 (m, 4H), 6.98–6.89 (m, 6H), 5.03 (s, 1H), 4.79 (s, 2H). ^13^C{^1^H} NMR (101 MHz, CDCl_3_) δ 170.9, 160.6, 158.2, 147.9, 143.8 (d, *J* = 2.8 Hz), 137.2, 132.1, 130.7 (d, *J* = 7.8 Hz), 129.8, 126.6 (d, *J* = 8.0 Hz), 122.4, 122.0, 116.6 (d, *J* = 22.6 Hz), 95.0, 74.7, 55.9. HRMS (APCl) calcd for C_28_H_19_BrCl_3_F_2_NO_2_ [M + H]^+^ 623.9705, found, 623.9708. IR (neat): 2954, 1750, 1604,1498,1406, 1369, 1311, 1276, 1215, 1188, 1127, 1093, 1073, 1010, 906, 829, 797 763, 728, 671, 649, 632, 564, 516 cm^–1^.

#### 2,2,2-Trichloroethyl-2-(4-(bis(4-bromophenyl)amino)phenyl)-2-(4-bromophenyl)acetate (**5b**)

The material was purified by flash chromatography (SiO_2_; hexanes/EtOAc, 10:0 to 9:1 gradient; *R*_f_ = 0.58 in 9:1 hexanes/EtOAc), which afforded **5b** as a colorless gel (134 mg, 90% yield) (1.55 g, 84% yield for one gram-scale). ^1^H NMR (600 MHz, CDCl_3_) δ 7.51 (d, *J* = 8.6 Hz, 2H), 7.36 (d, *J* = 8.8 Hz, 4H), 7.28 (d, *J* = 8.2 Hz, 4H), 7.22 (d, *J* = 8.2 Hz, 2H), 7.02 (d, *J* = 8.6 Hz, 2H), 6.94 (d, *J* = 8.8 Hz, 4H), 5.07 (s, 1H), 4.82 (s, 2H). ^13^C{^1^H} NMR (151 MHz, CDCl_3_) δ 170.8, 146.8, 146.5, 137.0, 132.8, 132.3, 132.2, 130.7, 130.0, 126.1, 124.3, 122.1, 116.3, 95.0, 74.7, 56.0. HRMS (APCl) calcd for C_28_H_19_Br_3_Cl_3_NO_2_ [M + H]^+^ 743.8104, found, 743.8107. IR (neat): 3032, 2952, 1750, 1607, 1507,1578, 1507, 1482, 1406, 1369, 1311, 1270, 1177, 1128, 1071, 1010, 906, 820, 765, 730, 669, 649, 571, 537, 510, 458 cm^–1^.

#### 2,2,2-Trichloroethyl-2-(4-(bis(4-methoxyphenyl)amino)phenyl)-2-(4-bromophenyl) acetate (**5c**)

The material was purified by flash chromatography (SiO_2_; hexanes/EtOAc, 9:1 to 7:3 gradient; *R*_f_ = 0.83 in 7:3 hexanes/EtOAc), which afforded **5c** as a colorless gel (118 mg, 91% yield). ^1^H NMR (600 MHz, CDCl_3_) δ 7.47 (d, *J* = 8.5 Hz, 2H), 7.25 (d, *J* = 8.5 Hz, 2H), 7.09 (d, *J* = 8.8 Hz, 2H), 7.03 (d, *J* = 7.8 Hz, 4H), 6.86 (d, *J* = 8.8 Hz, 2H), 6.81 (d, *J* = 7.8 Hz, 4H), 5.02 (s, 1H), 4.79 (q, *J* = 11.9 Hz, 2H), 3.79 (s, 6H). ^13^C{^1^H} NMR (101 MHz, CDCl_3_) δ 171.1, 156.3, 148.7, 141.0, 137.5, 132.1, 130.7, 129.4, 128.8, 127.1, 121.9, 120.5, 115.0, 95.0, 74.7, 55.8 (two peaks separated by 0.03 ppm). HRMS (APCl) calcd for C_30_H_25_BrCl_3_NO_4_ [M + H]^+^ 648.0124, found, 648.0099. IR (neat): 2952, 2834, 1750, 1605, 1501, 1463, 1440, 1369, 1319, 1274, 1237, 1179, 1127, 1073, 1033, 1011, 905, 827, 780, 762, 726, 648, 571, 525, 502 cm^–1^.

#### 2,2,2-Trichloroethyl-2-(4-bromophenyl)-2-(4-(di-p-tolylamino)phenyl)acetate (**5d**)

The material was purified by flash chromatography (SiO_2_; hexanes/EtOAc, 10:0 to 9:2 gradient; *R*_f_ = 0.56 in 9:1 hexanes/EtOAc), which afforded **5d** as a colorless foam (106 mg, 86% yield). ^1^H NMR (400 MHz, CDCl_3_) δ 7.47 (d, *J* = 8.5 Hz, 2H), 7.26 (d, *J* = 8.5 Hz, 2H), 7.13 (d, *J* = 8.6 Hz, 2H), 7.06 (d, *J* = 8.3 Hz, 4H), 6.98–6.94 (m, 6H), 5.03 (s, 1H), 4.80 (q, *J* = 11.9 Hz, 2H), 2.31 (s, 6H). ^13^C{^1^H} NMR (101 MHz, CDCl_3_) δ 171.1, 148.1, 145.4, 137.4, 133.1, 132.1, 130.7, 130.3, 130.0, 129.5, 125.1, 122.5, 121.9, 95.0, 74.7, 55.9, 21.2. HRMS (APCl) calcd for C_30_H_25_BrCl_3_NO_2_ [M + H]^+^ 616.0207, found, 616.0209. IR (neat): 3026, 2919, 2245, 1750, 1604, 1504, 1488, 1406, 1369, 1319, 1292, 1271, 1211, 1179, 1125, 1073, 1011, 906, 814, 762, 728, 648, 568, 511, 457 cm^–1^.

#### 2,2,2-Trichloroethyl-2-(4-bromophenyl)-2-(4-(diphenylamino)phenyl)acetate (**5e**) and tris(2,2,2-trichloroethyl)-2,2′,2″-(nitrilotris(benzene-4,1-diyl))tris(2-(4-bromophenyl) acetate) (**5f**)

**5e** as major product (60%) and **5e** as minor product (17%). The material was purified by flash chromatography (SiO_2_; hexanes/EtOAc, 10:0 to 9:1 gradient; *R*_f_ = 0.53 in 9:1 hexanes/EtOAc), which afforded major product **5e** as a colorless gel (70 mg, 60%): ^1^H NMR (400 MHz, CDCl_3_) δ 7.49 (d, *J* = 8.4 Hz, 2H), 7.31–7.21 (m, 6H), 7.18 (d, *J* = 6.7 Hz, 2H), 7.08 (d, *J* = 7.5 Hz, 4H), 7.06–6.99 (m, 4H), 5.05 (s, 1H), 4.81 (t, *J* = 1.7 Hz, 2H). ^13^C{^1^H} NMR (101 MHz, CDCl_3_) δ 171.0, 148.2, 147.8, 147.8, 137.2, 132.1, 131.0, 130.7, 129.7, 129.6, 129., 124.9, 124.5, 123.7, 123.5, 123.0, 122.0, 95.0, 74.7, 56.0. HRMS (ESI) calcd for C_28_H_21_BrCl_3_NO_2_ [M + H]^+^ 587.9894, found, 587.9901. IR (neat): 3034, 2952, 1751, 1588, 1507, 1487, 1449, 1406, 1369, 1329, 1313, 1275, 1177, 1127, 1073, 1027, 1011, 908, 830, 753, 731, 695, 636, 621, 571, 531, 510, 449, 426 cm^–1^. The material was purified by flash chromatography (SiO_2_; hexanes/EtOAc, 10:0 to 9:1 gradient; *R*_f_ = 0.39 in 9:1 hexanes/EtOAc), which afforded minor product **5f** as a colorless foam (43 mg, 17%): ^1^H NMR (600 MHz, CDCl_3_) δ 7.47 (d, *J* = 8.6 Hz, 6H), 7.25 (d, *J* = 8.6 Hz, 6H), 7.17 (d, *J* = 8.6 Hz, 6H), 6.99 (d, *J* = 8.6 Hz, 6H), 5.04 (s, 3H), 4.79 (s, 6H). ^13^C{^1^H} NMR (101 MHz, CDCl_3_) δ 170.9, 147.1, 137.1, 132.2, 132.1, 130.7, 129.9, 124.6, 122.1, 95.0, 74.7, 56.0. HRMS (ESI) calcd for C_48_H_33_Br_3_Cl_9_NO_6_ [M + H]^+^ 1270.7022, found, 1270.7027. IR (neat): 3031, 2953, 1749, 1602, 1505, 1487, 1445, 1406, 1369, 1323, 1275, 1180, 1127, 1073, 1011, 907, 831, 763, 717, 649, 571, 536, 501, 465 cm^–1^. When an excess amount of diazo derivative (3.5 equiv) was used, the major product of **4e** is **5f** in an isolated yield of 70% and the minor product is **5e** in an isolated yield of 14%.

#### Tetrakis(2,2,2-trichloroethyl)-2,2′,2″,2‴-(([1,1′-biphenyl]-4,4′-diylbis(azanetriyl))tetrakis (benzene-4,1-diyl))tetrakis(2-(4-bromophenyl) acetate) (**7**)

The material was purified by flash chromatography (SiO_2_; hexanes/EtOAc, 10:0 to 9:1 gradient; *R*_f_ = 0.55 in 9:1 hexanes/EtOAc), which afforded **7** as a colorless foam (226 mg, 61% yield using 4.5 equiv diazo derivative). ^1^H NMR (600 MHz, CDCl_3_) δ 7.48 (d, *J* = 8.5 Hz, 8H), 7.43 (d, *J* = 8.6 Hz, 4H), 7.27 (d, *J* = 8.5 Hz, 8H), 7.19 (d, *J* = 8.6 Hz, 8H), 7.09 (d, *J* = 8.6 Hz, 4H), 7.05 (d, *J* = 8.6 Hz, 8H), 5.05 (s, 4H), 4.80 (s, 8H). ^13^C{^1^H} NMR (151 MHz, CDCl_3_) δ 170.9, 147.3, 146.5, 137.1, 135.6, 132.2, 131.8, 130.7, 129.8, 127.8, 125.0, 124.4, 122.1, 95.0, 74.7, 56.0. HRMS (ESI) calcd for C_76_H_52_Br_4_Cl_12_N_2_O_8_ [M + H]^+^ 1855.67140, found, 1855.67295. IR (neat): 3031, 2953, 2250, 1903, 1748, 1600, 1505, 1488, 1406, 1369, 1321, 1273, 1179, 1126, 1073, 1010, 905, 824, 762, 726, 649, 571, 529, 502, 464.

### Synthesis of *N*-(4-Iodophenyl)-*cis*-5-norbornene-*exo*-2,3-dicarboxylic Imide (**10**)

To a 40 mL toluene solution of **8** (2.0 g, 12.2 mmol) were added 4-iodoaniline **9** (4.0 g, 18 mmol) and Et_3_N (2.0 mL). The resulting mixture was allowed to stir at reflux (achieving using a heating block containing sand) for 15 h. The reaction mixture was cooled down to room temperature, and a white precipitate was formed. The solid was filtered, rinsed with toluene, and dried to get the desired product as white crystals (3.0 g, 8.0 mmol), 67%. ^1^H NMR (500 MHz, CDCl_3_) δ 7.79 (d, *J* = 8.6 Hz, 2H), 7.04 (d, *J* = 8.6 Hz, 2H), 6.35 (t, *J* = 1.9 Hz, 2H), 3.40 (t, *J* = 1.8 Hz, 2H), 2.85 (d, *J* = 1.3 Hz, 2H), 1.62 (d*t* = 9.9, 1.6 Hz, 1H), 1.43 (d, *J* = 9.9 Hz, 1H). ^13^C{^1^H} NMR (126 MHz, CDCl_3_) δ 176.8, 138.5, 138.1, 131.6, 128.2, 94.2, 48.0, 46.0, 43.1. HRMS (ESI) calcd for C_15_H_13_INO_2_ [M + H]^+^ 365.9985, found, 365.9991. Calcd for C_15_H_12_INO_2_: C, 49.34; H, 3.31; N, 3.84. Found: C, 49.13; H, 3.23; N, 3.88.

### Synthesis of Norbornene-Azoacetate Derivative **11**

Pd(PPh_3_)_4_ (664 mg, 0.574 mmol, 10 mol %), PPh_3_ (300 mg, 1.14 mmol, 20 mol %), compound **10** (2.1 g, 5.75 mmol, 1.0 equiv), and Ag_2_CO_3_ (792 mg, 2.87 mmol, 0.5 equiv) was suspended in toluene (25.0 mL) under nitrogen, followed by addition of NEt_3_ (1.16 g, 11.5 mmol, 2.0 equiv) and ethyl diazoacetate (1.31 g, 11.5 mmol, 2.0 equiv). The resulting reaction was stirred at room temperature overnight and then filtered through a short path of silica gel, eluting with ethyl acetate. The volatile compounds were removed in vacuo, and the residue was purified by column chromatography (3:2 Hex/EtOAc) to give the product as a yellow solid (1.0 g, 2.8 mmol), 50%. ^1^H NMR (500 MHz, CDCl_3_) δ 7.60–7.53 (m, 2H), 7.33–7.27 (m, 2H), 6.35 (t, *J* = 1.9 Hz, 2H), 4.34 (q, *J* = 7.2 Hz, 2H), 3.41 (t, *J* = 1.8 Hz, 2H), 2.86 (d, *J* = 1.3 Hz, 2H), 1.62 (dt, *J* = 9.8, 1.6 Hz, 1H), 1.48 (d, *J* = 9.9 Hz, 1H), 1.34 (t, *J* = 7.1 Hz, 3H). ^13^C{^1^H} NMR (126 MHz, CDCl_3_) δ 177.2, 164.9, 138.2, 129.3, 126.9, 126.6, 124.5, 63.7, 61.3, 48.0, 46.0, 43.2, 14.6. HRMS (ESI) calcd for C_19_H_21_N_4_O_4_ [M + NH_4_]^+^: 369.1557, found, 369.1560. Calcd for C_19_H_17_N_3_O_4_: C, 64.95; H, 4.88; N, 11.96. Found: C, 64.48; H, 4.98; N, 10.88 (we were unable to obtain analysis within acceptable limits despite several attempts).

### Synthesis of Norbornene-Bis(diarylamino)biphenyl Monomer, **M1**

In a 20 mL vial with a stir bar, **12** (100 mg, 0.182 mmol) and Rh_2_(*S*-DOSP)_4_ (4.2 mg, 0.0022 mmol, 1.2 mol %) were added, capped, evacuated, and refilled with nitrogen three times. Anhydrous CH_2_Cl_2_ (1.0 mL added) was used to dissolve all of the solids. In another vial, **11** (92 mg, 0.26 mmol) was added, evacuated, and refilled three times. Dry CH_2_Cl_2_ (5.0 mL) was added to dissolve all solids, and then the yellow solution was taken into a syringe and added dropwise to the vial containing MeO-TPD over 2 h. The reaction mixture was stirred overnight at reflux (achieved using a heating block containing sand). The solvent was evaporated under reduced pressure to yield a crude product, in which mass spectrometry indicated the presence of both the desired compound and a minor product identified as a double-insertion product. The desired product, **M1**, was purified by column chromatography (3:2 hexane/EtOAc) and obtained as a pale yellow solid (85 mg, 0.075 mmol), 53%. ^1^H NMR (700 MHz, CDCl_3_) δ 7.47 (d, *J* = 8.5 Hz, 2H), 7.43–7.36 (d, *J* = 8.3, 4H), 7.25–7.20 (m, 4H), 7.16 (d, *J* = 8.3 Hz, 2H), 7.12–7.03 (m, 10H), 6.99 (d, *J* = 8.2 Hz, 2H), 6.95 (m, 1H), 6.85 (dd, *J* = 9.0, 3.6 Hz, 4H), 6.35 (m, 2H), 4.93 (s, 1H), 4.20 (q, *J* = 7.1 Hz, 2H), 3.81 (s, 3H), 3.80 (s, 3H), 3.40 (m, 2H), 2.85 (d, *J* = 1.4 Hz, 2H), 1.61 (d, *J* = 9.9, 1.6 Hz, 1H), 1.47 (d, *J* = 9.9, 1.6 Hz, 1H), 1.27 (t, *J* = 7.1 Hz, 3H). ^13^C{^1^H} NMR (176 MHz, CDCl_3_) δ 177.2, 172.3, 171.3, 156.5, 156.3, 148.2, 147.4, 147.1, 146.8, 140.8, 140.5, 139.7, 138.1, 134.4, 134.2, 131.5, 130.9, 129.5, 129.3, 129.2, 127.6, 127.5, 127.3, 123.3, 123.2, 123.0, 122.7, 122.1, 114.9 (two peaks separated by 0.03 ppm), 61.5, 60.5, 56.3, 55.6, 48.0, 46.0, 43.1, 21.2, 14.3, 14.3. HRMS (ESI) calcd for C_57_H_50_N_3_O_6_ [M + H]^+^, 872.3694; found, 872.3678. [Minor double-insertion product (not isolated pure): ERMS(EI) calcd C_76_H_67_N_4_O_10_ [M + H]^+^, 1195.4852; found, 1195.4832.]

### Synthesis of Poly(norbornene-bis(diarylamino)biphenyl) **P1**

Monomer **M1** (66 mg, 0.07 mmol) was dissolved in anhydrous and deoxygenated CH_2_Cl_2_ (600 μL). Grubbs third-generation initiator (0.7 mg, 0.0008 mmol, 1 mol %) was dissolved in anhydrous and deoxygenated CH_2_Cl_2_ (200 μL) and added quickly to the stirring monomer solution. The solution was stirred at room temperature under nitrogen for 20 min before ethyl vinyl ether (100 μL) was added; the mixture was then stirred for 5 min. The polymer was precipitated in MeOH (4 mL) and reprecipitated twice from CH_2_Cl_2_ and MeOH to yield a yellow powder (40 mg, 60%). ^1^H NMR (700 MHz, CDCl_3_) δ 7.47–7.27 (br, 5H), 7.24–6.64 (br, 24H), 5.84–5.61 (br, 1H), 5.60–7.33 (br, 1H), 4.98–4.62 (br, 1H), 7.23–4.01 (br, 2H), 3.84–3.67 (br, 6H), 3.62–3.34 (br, 1H), 3.34–2.94 (br, 3H), 2.94–2.57 (br, 1H), 2.34–1.95 (br, 1H), 2.36–2.00 (br, 1H), 1.71–1.43 (br, 2H), 1.30–1.00 (m, 3H). *M*_w_ = 150 kDa, PDI = 1.2 (determined via GPC in CHCl_3_). Anal. Calcd for (C_57_H_49_N_3_O_6_)_n_: C, 78.69; H, 5.45; N, 4.83. Found: C, 77.37; H, 5.66; N, 4.88.

## Data Availability

The data underlying this study are available in the published article and its Supporting Information.

## Supporting Information Available

Summary of diazo derivatives and triarylamine structures studied, NMR spectra of new compounds, and HPLC traces (PDF)
