# Supplementary material for: Dirhodium C–H Functionalization of Hole-Transport Materials
Source: J Org Chem. 2023 Mar 15;88(7):4309–16. doi: 10.1021/acs.joc.2c02888 (PMC10088024; doi:10.1021/acs.joc.2c02888)
Supplement: Supplementary file 1 — jo2c02888_si_001.pdf [file jo2c02888_si_001.pdf]

Dirhodium C-H Functionalization of Hole-Transport Materials

Supporting Information

(27 Pages)

Farzaneh Saeedifard,[a,b] Yasir Naeem,[c] Yannick T. Boni,[c] Yi-Chien Chang,[d] Junxiang Zhang,[b] Yadong Zhang,[b] Bernard Kippelen,[d] Stephen Barlow,[a,b] Huw M. L. Davies,*[,c] Seth R. Marder*[,a,b, e,f,g]

[a] School of Chemistry and Biochemistry, Georgia Institute of Technology, Atlanta, GA 30332, USA, [b] Renewable and Sustainable Energy Institute, University of Colorado Boulder, Boulder, CO 80303, USA, [c] Department of Chemistry, Emory University, Atlanta, Georgia 30322, United States, [d] School of Electrical and Computer Engineering, Center for Organic Photonics and Electronics (COPE), Georgia Institute of Technology, Atlanta, GA 30332, USA, [e] Department of Chemical and Biological Engineering, University of Colorado Boulder, Boulder, CO 80303, USA, [f] Materials Science and Engineering Program, University of Colorado Boulder, Boulder, CO 80303, USA, [g] Department of Chemistry, University of Colorado Boulder, Boulder, CO 80303, USA

<u>Table of Contents</u>

Section 1: Diazo and Triarylamine Structures Utilized in This Study…..……………..…….S-1

Section 2: ^{1}H NMR and ^{13}C NMR Spectroscopic Data………………….……………….....S-2

Section 3: HPLC Traces……………………………………………..………………....S-27

SECTION 1: Diazo and Triarylamine Structures Utilized in this Study.

Chart S1. Azo Compounds Utilized in this Study

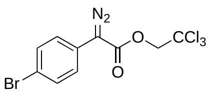

2

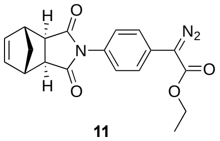

11

Chart S2. Triarylamines Utilized in this Study

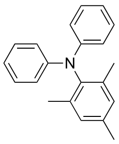

1

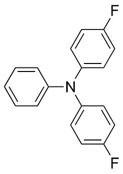

4a

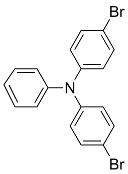

4b

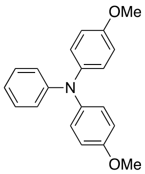

4c

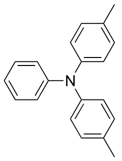

4d

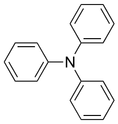

4e

Chart S3. Bis(diarylamino)biphenyls Utilized in this Study

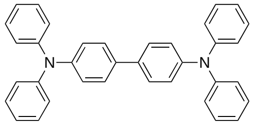

6

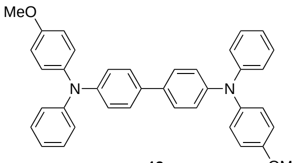

12

SECTION 2: ^{1}H and ^{13}C NMR Spectroscopic Data.

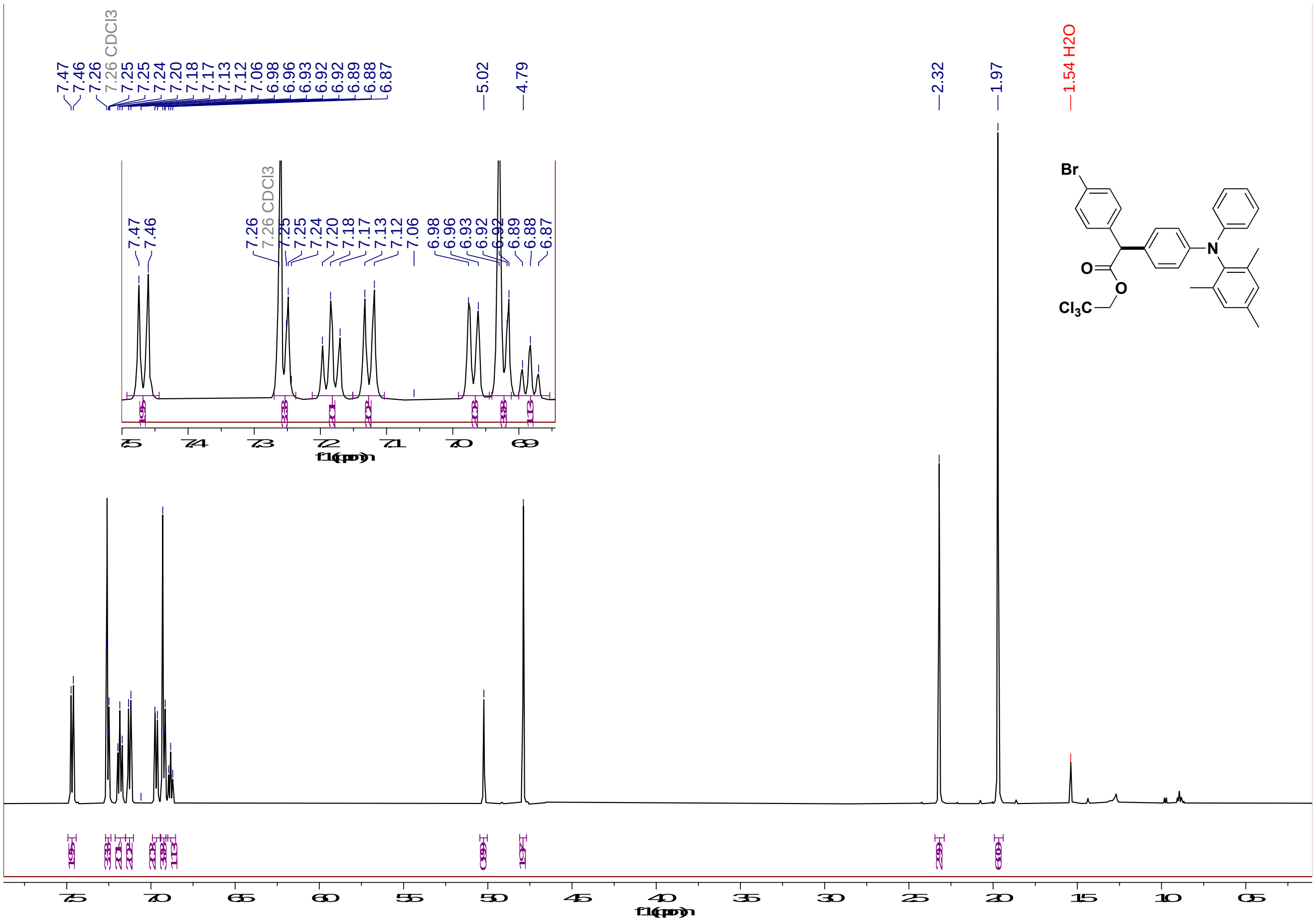

Figure S1. ^{1}H NMR spectrum (600 MHz, Chloroform-*d*) of **3a**.

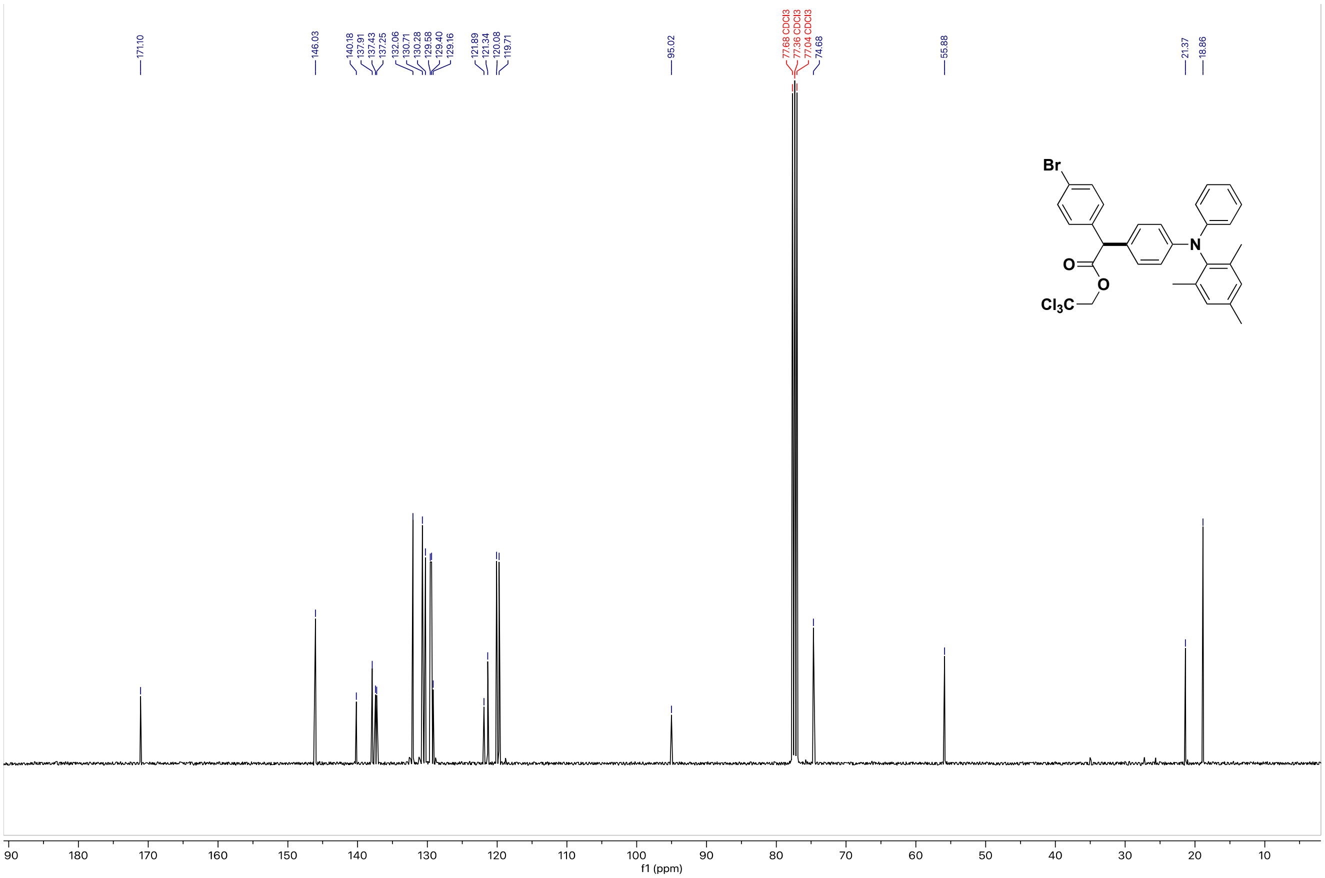

Figure S2. $^{13}C\{^{1}H\}$ NMR spectrum (101 MHz, Chloroform-*d*) of **3a**.

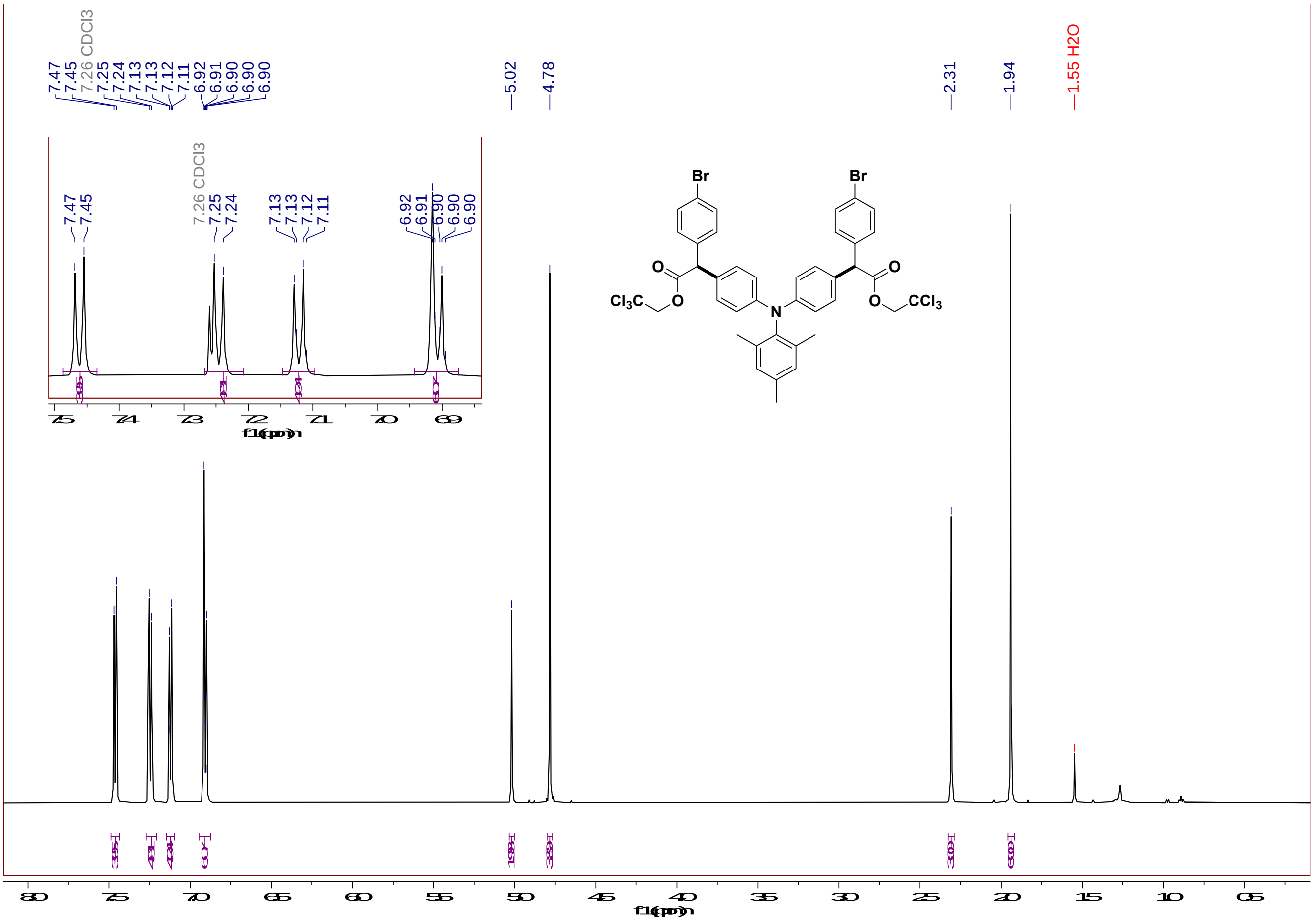

Figure S3. ^{1}H NMR spectrum (600 MHz, Chloroform-d) of **3b**.

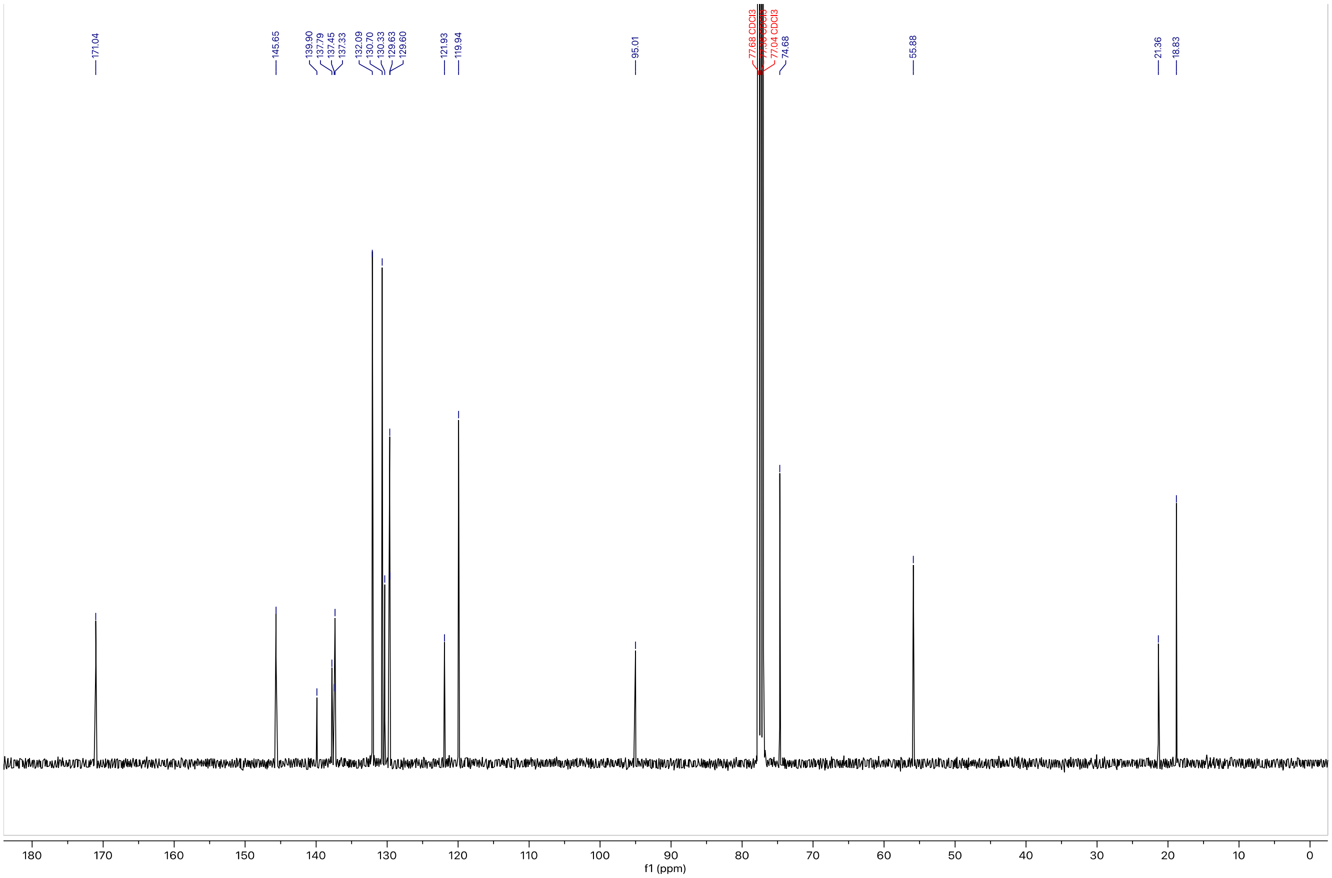

Figure S4. $^{13}C\{^{1}H\}$ NMR spectrum (101 MHz, Chloroform-*d*) of **3b**.

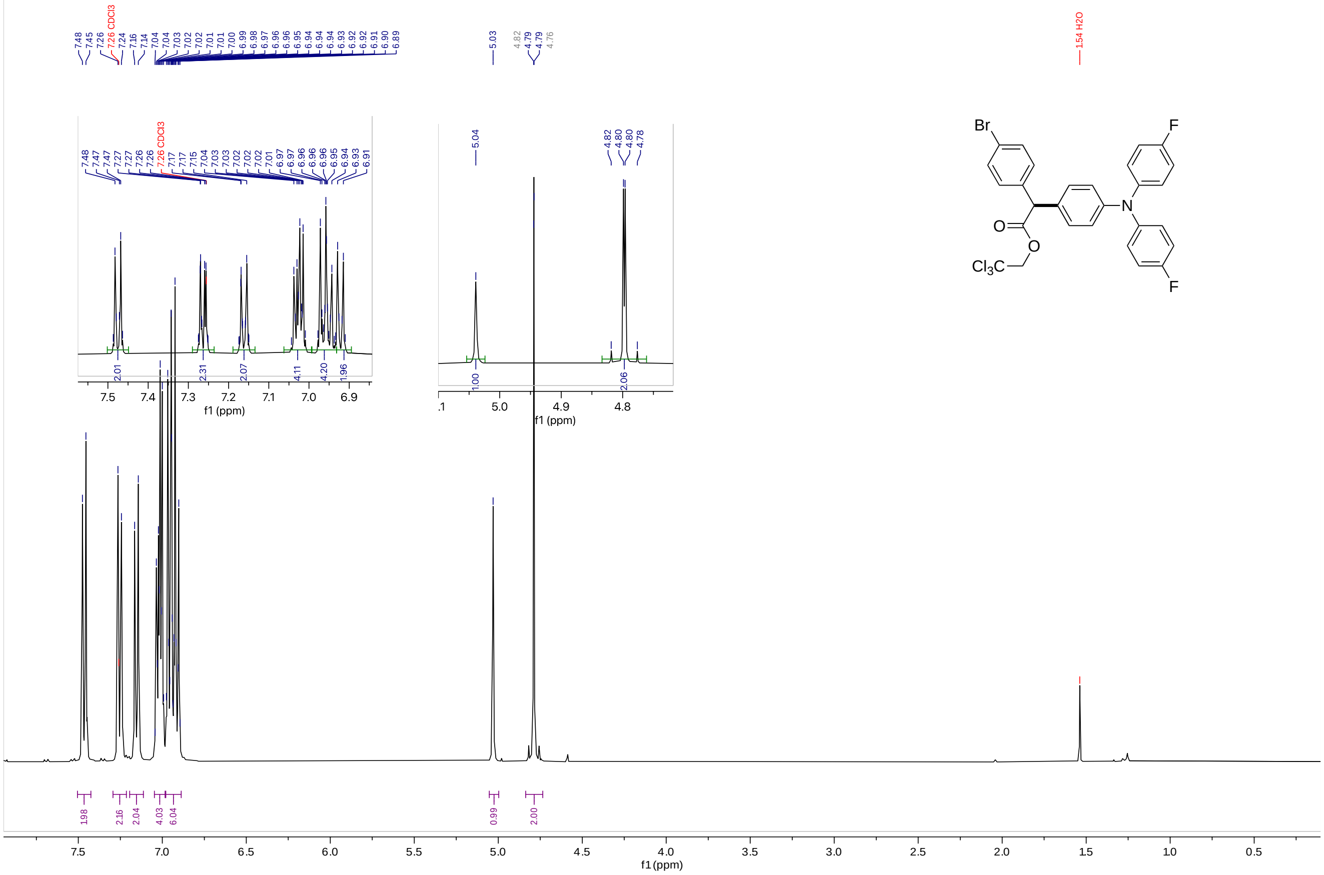

Figure S5. ^{1}H NMR spectrum (400 MHz, Chloroform-*d*) of **5a**.

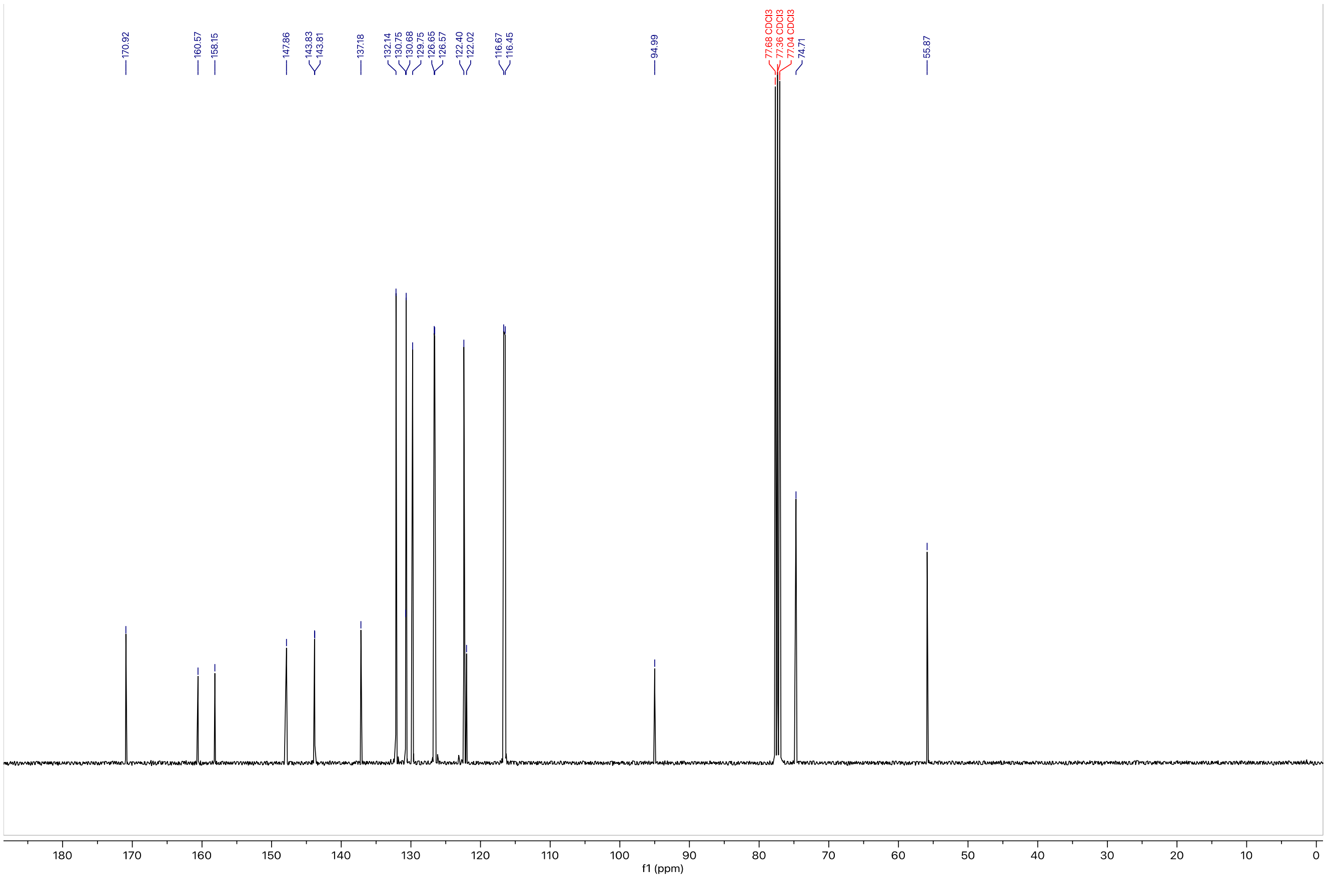

Figure S6. $^{13}C\{^{1}H\}$ NMR spectrum (101 MHz, Chloroform-*d*) of **5a**.

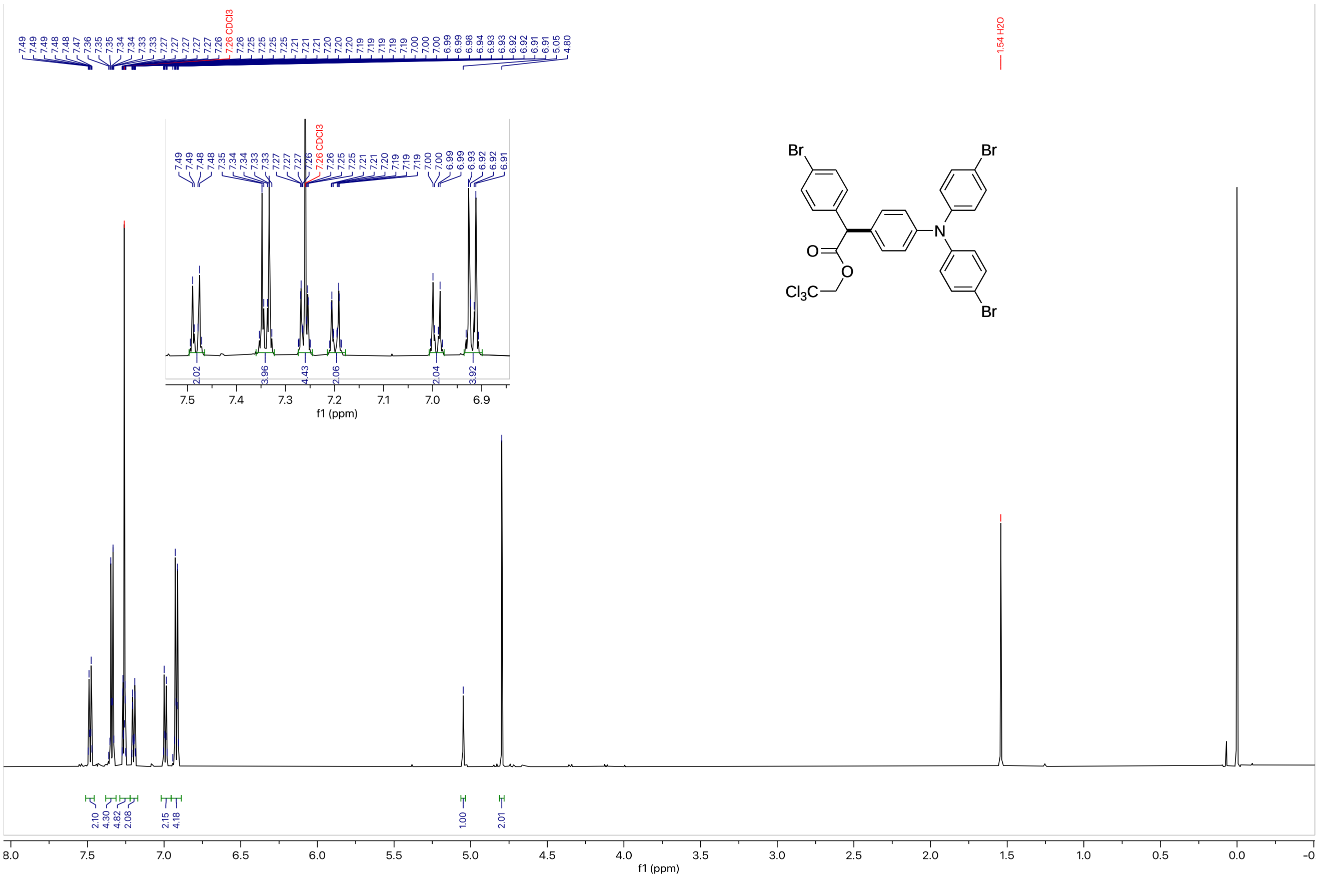

Figure S7. ^{1}H NMR spectrum (600 MHz, Chloroform-*d*) of **5b**.

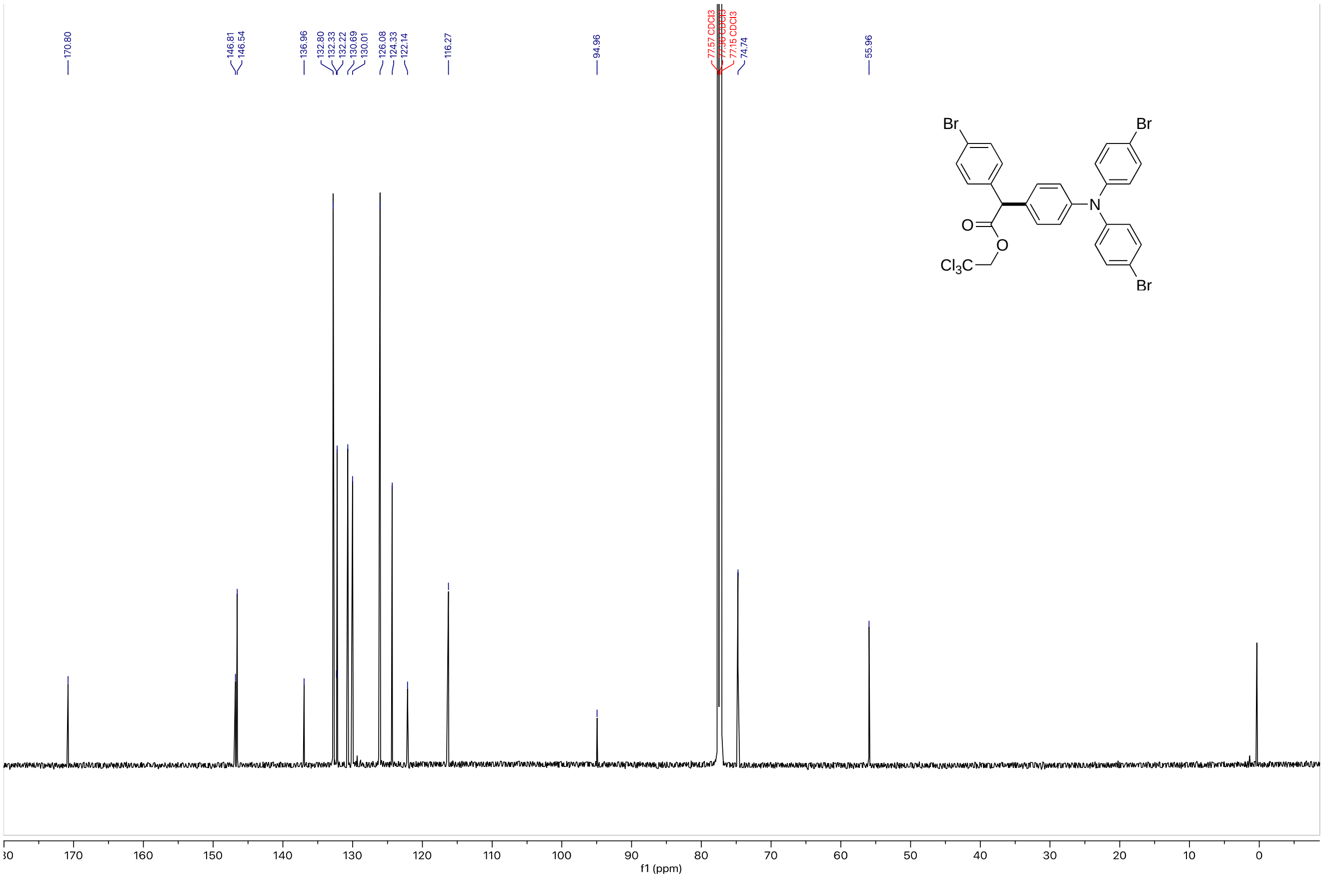

Figure S8. $^{13}C\{^{1}H\}$ NMR spectrum (151 MHz, Chloroform-*d*) of **5b**.

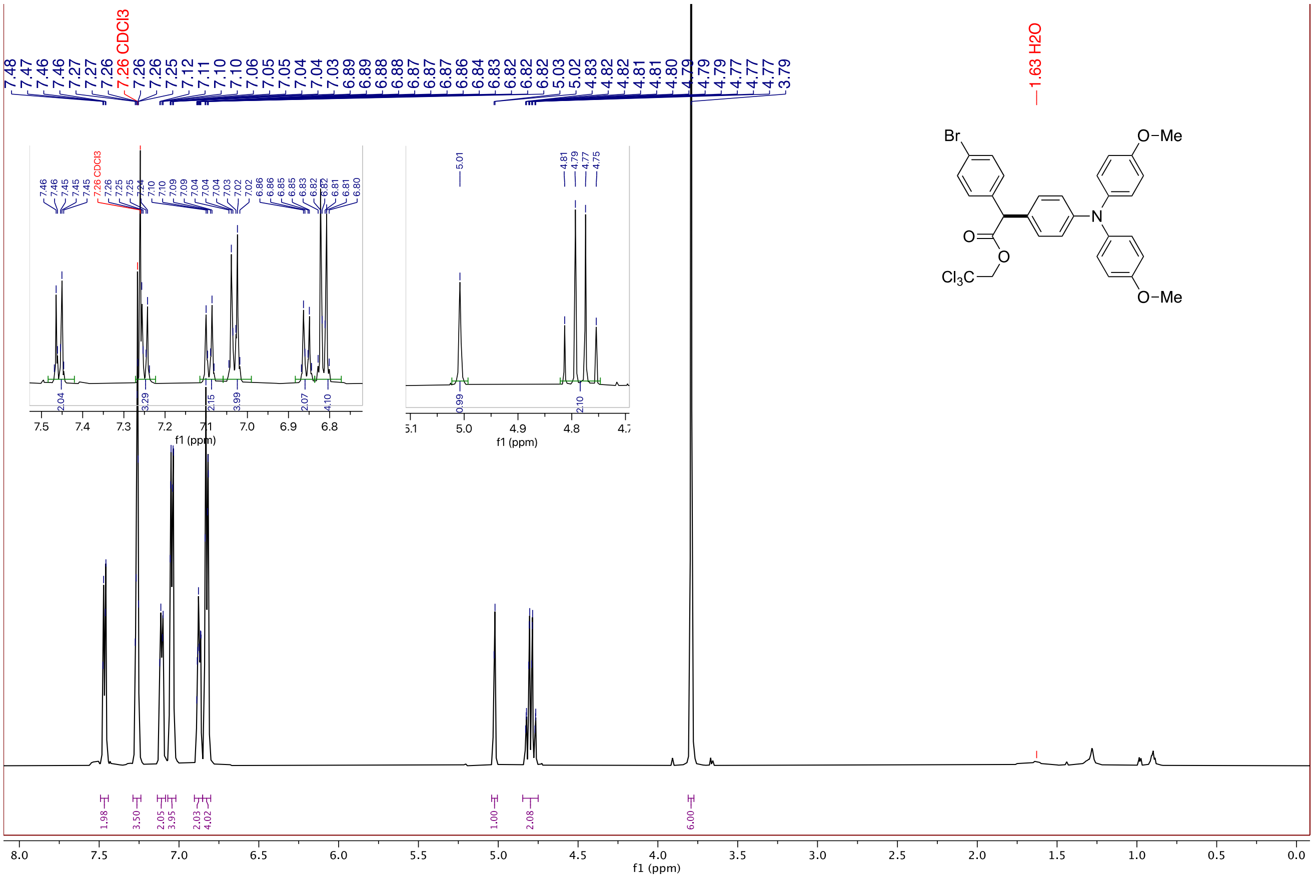

Figure S9. ^{1}H NMR spectrum (600 MHz, Chloroform-*d*) of **5c**.

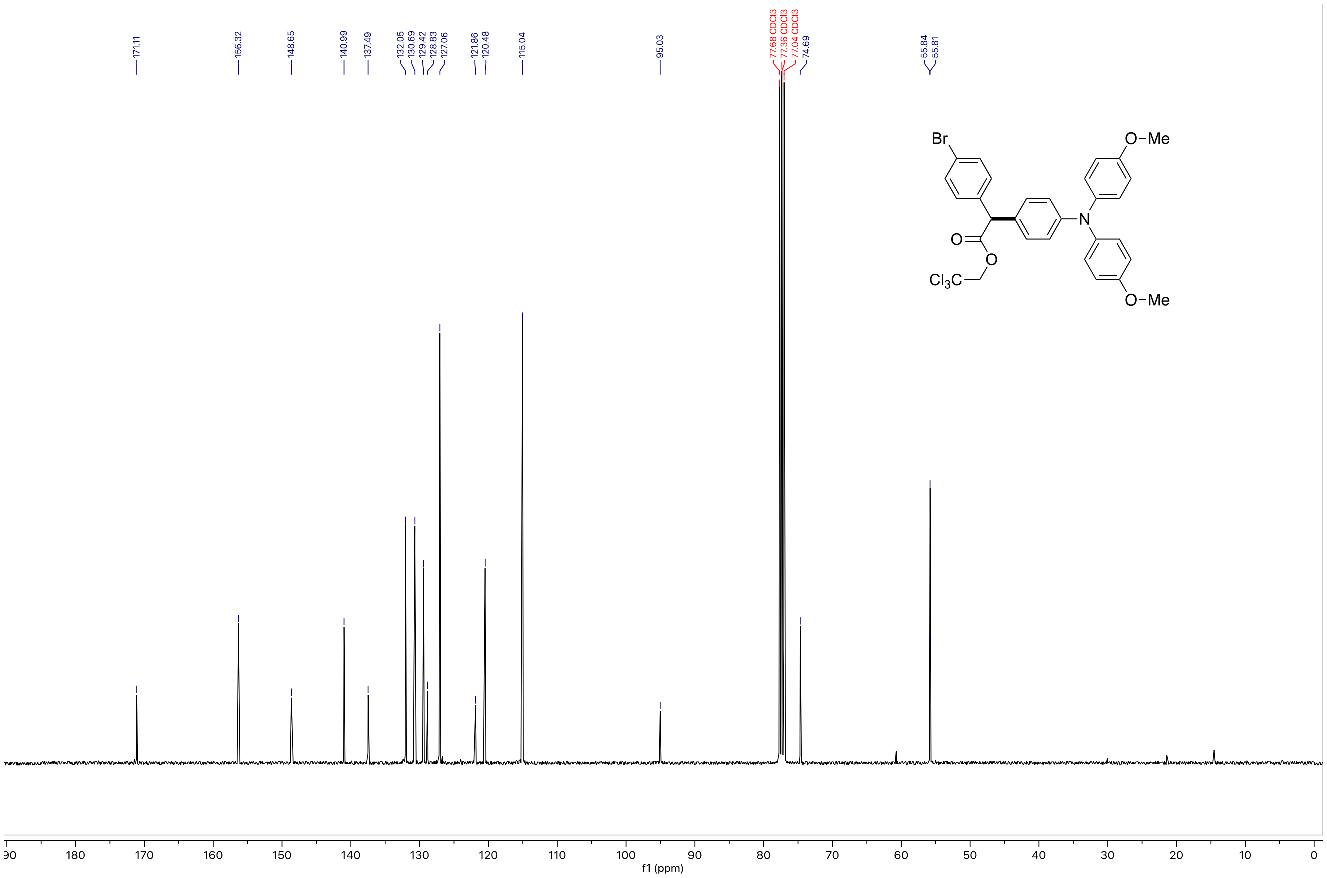

Figure S10. $^{13}C\{^{1}H\}$ NMR spectrum (101 MHz, Chloroform-*d*) of **5c**.

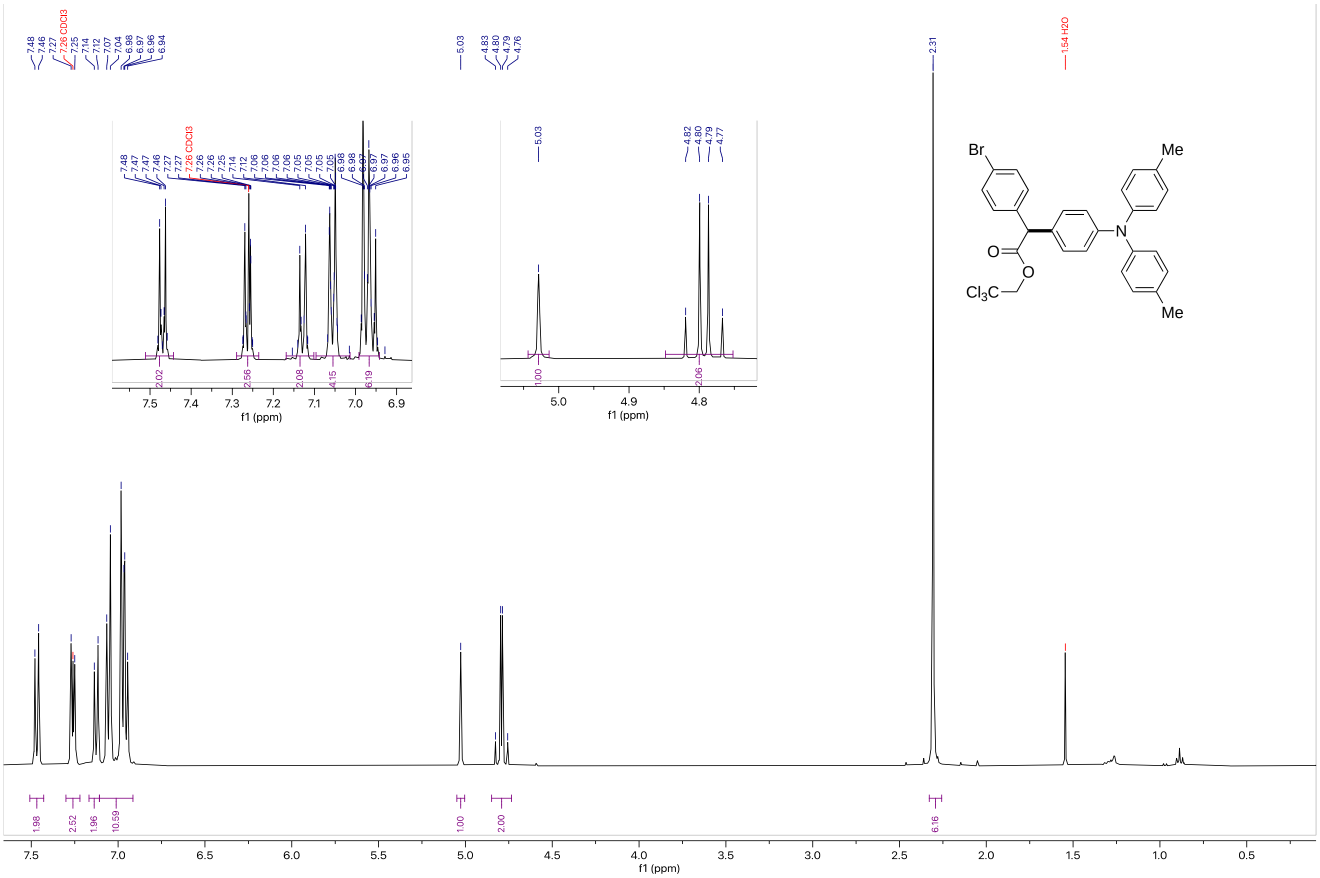

Figure 11. ^{1}H NMR spectrum (400 MHz, Chloroform-*d*) of **5d**.

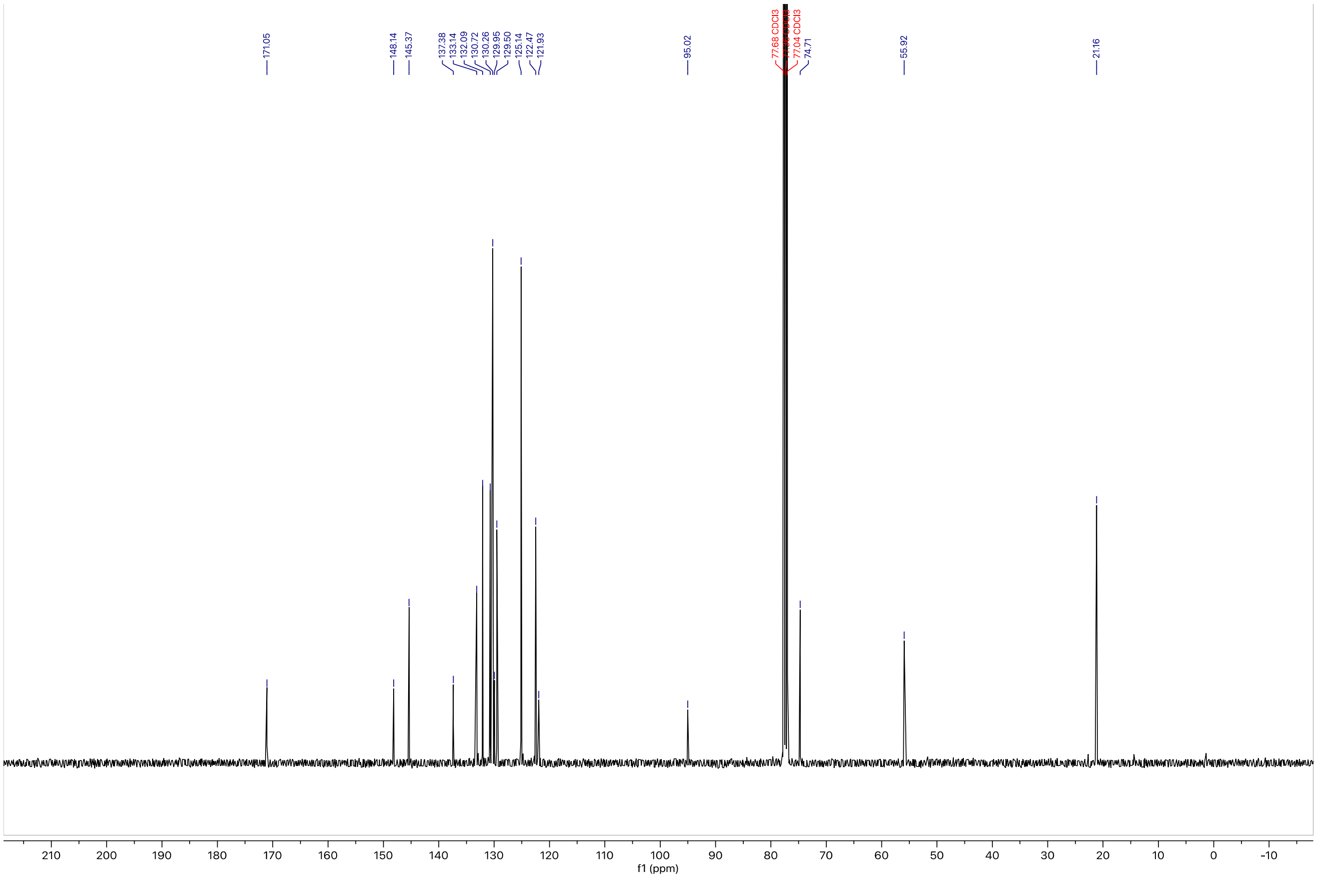

Figure S12. $^{13}C\{^{1}H\}$ NMR spectrum (101 MHz, Chloroform-*d*) of **5d**.

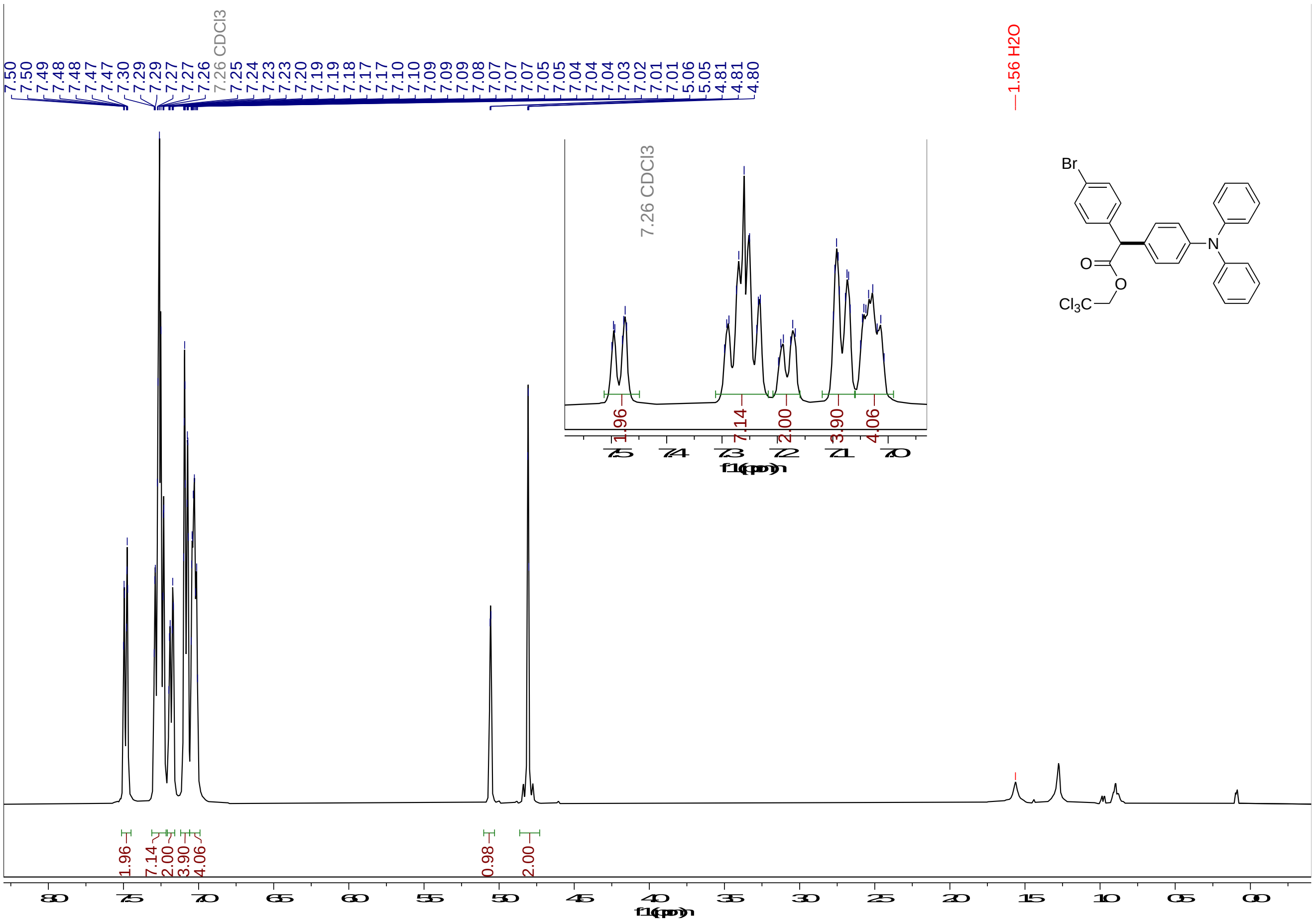

Figure S13. ^{1}H NMR spectrum (400 MHz, Chloroform-*d*) of **5e**.

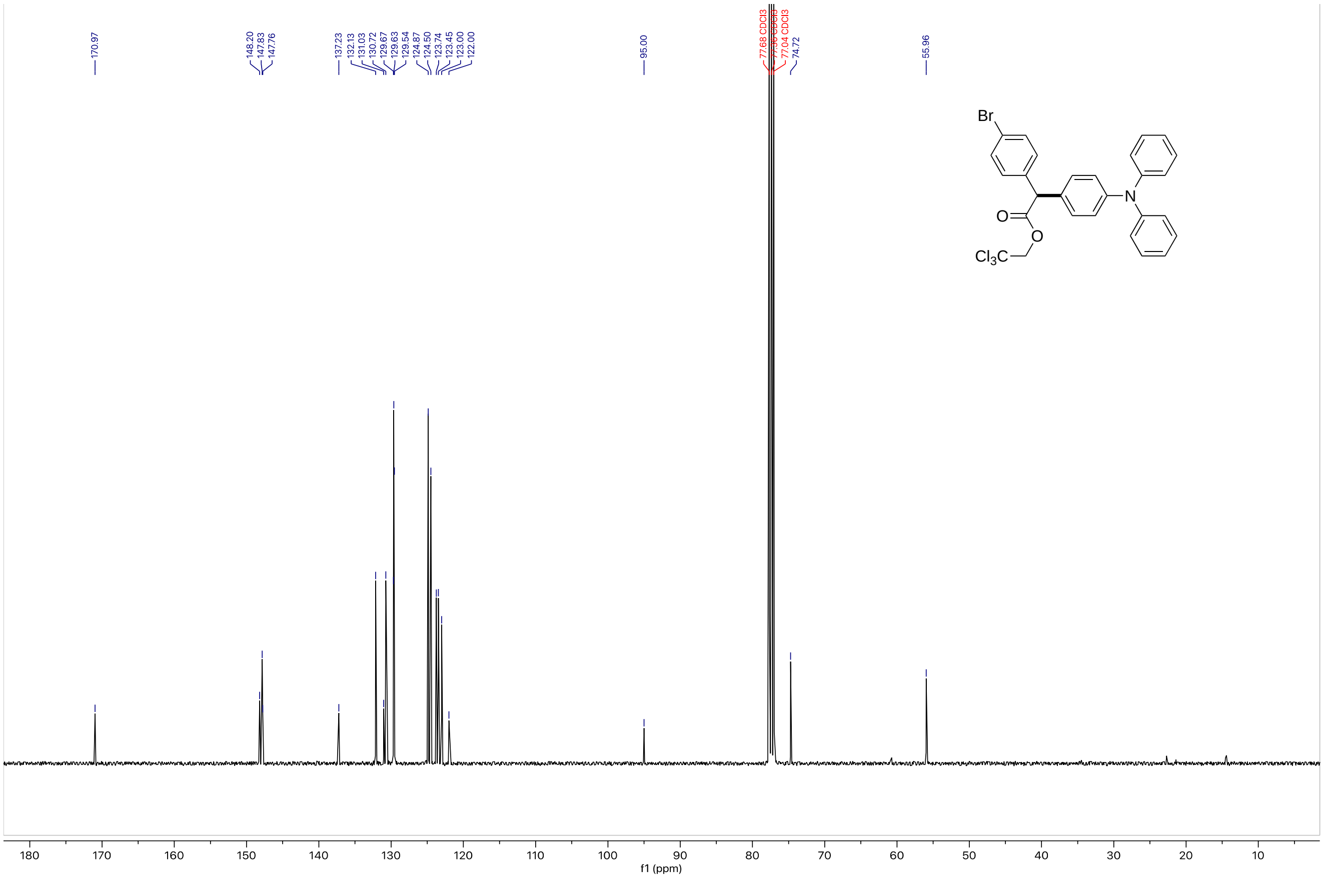

Figure S14. $^{13}C\{^{1}H\}$ NMR spectrum (101 MHz, Chloroform-*d*) of **5e**.

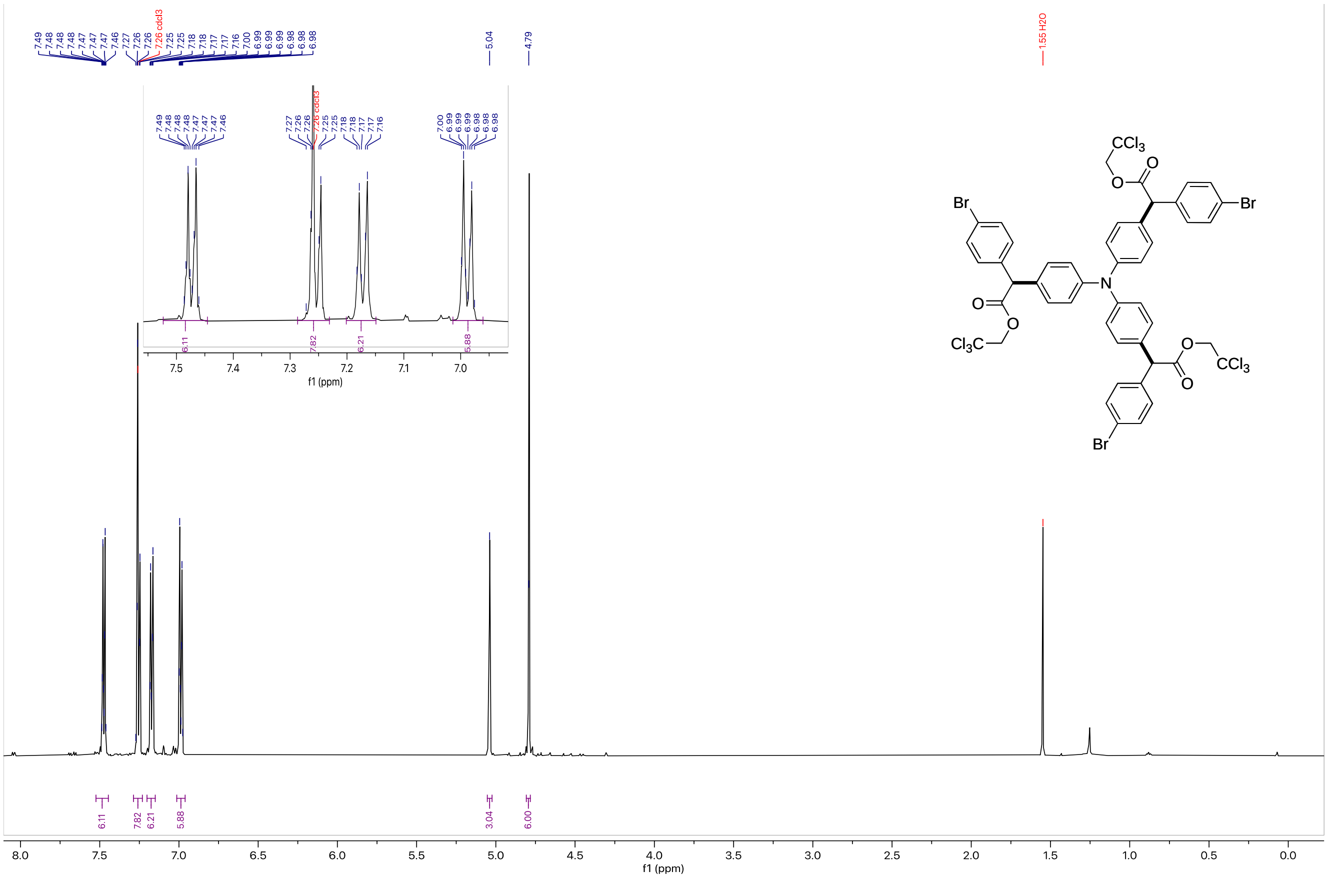

Figure S15. ^{1}H NMR spectrum (600 MHz, Chloroform-*d*) of **5f**.

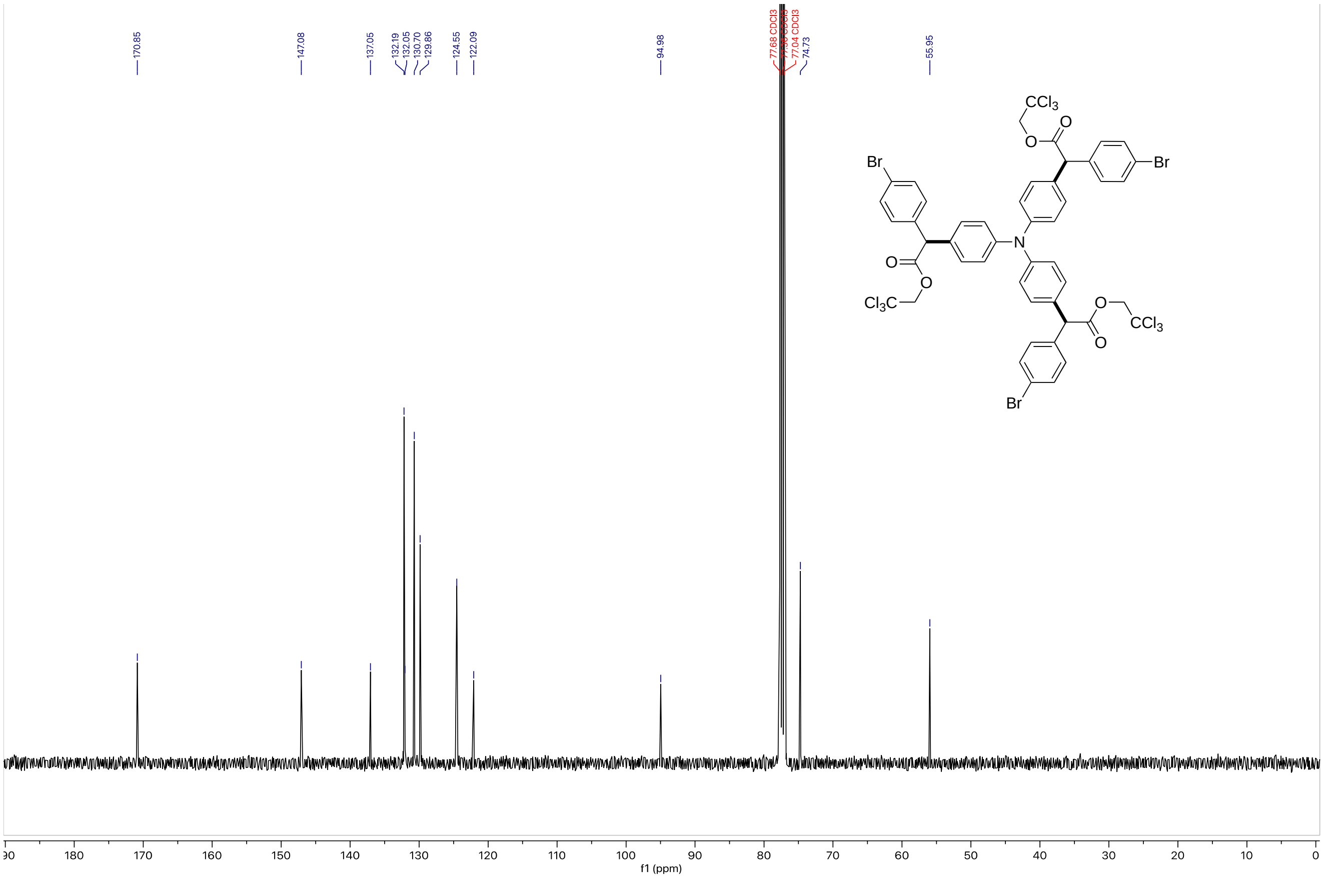

Figure S16. ^{13}C NMR spectrum (101 MHz, Chloroform-*d*) of **5f**.

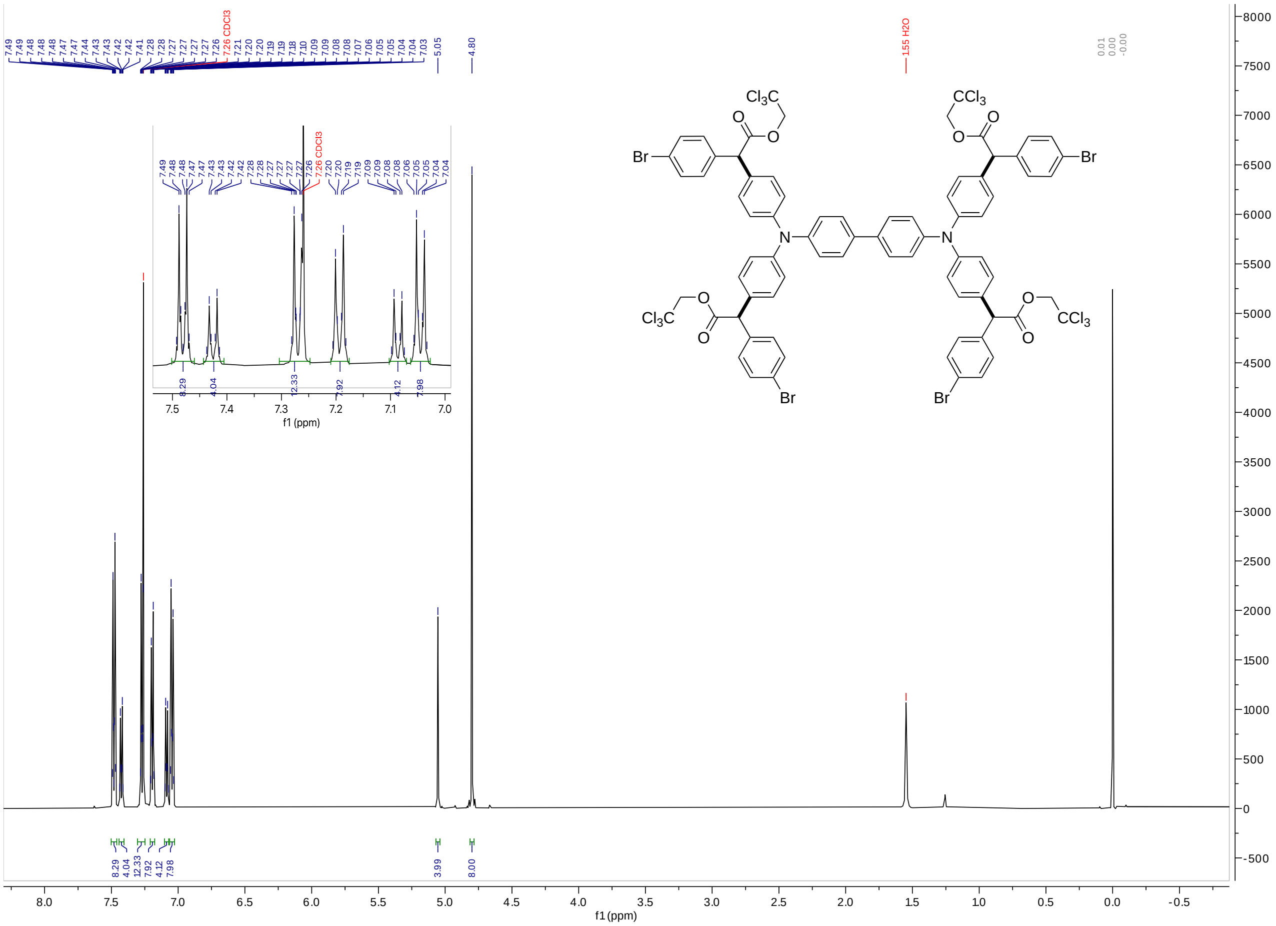

Figure S17. ^{1}H NMR spectrum (600 MHz, Chloroform-*d*) of **7**.

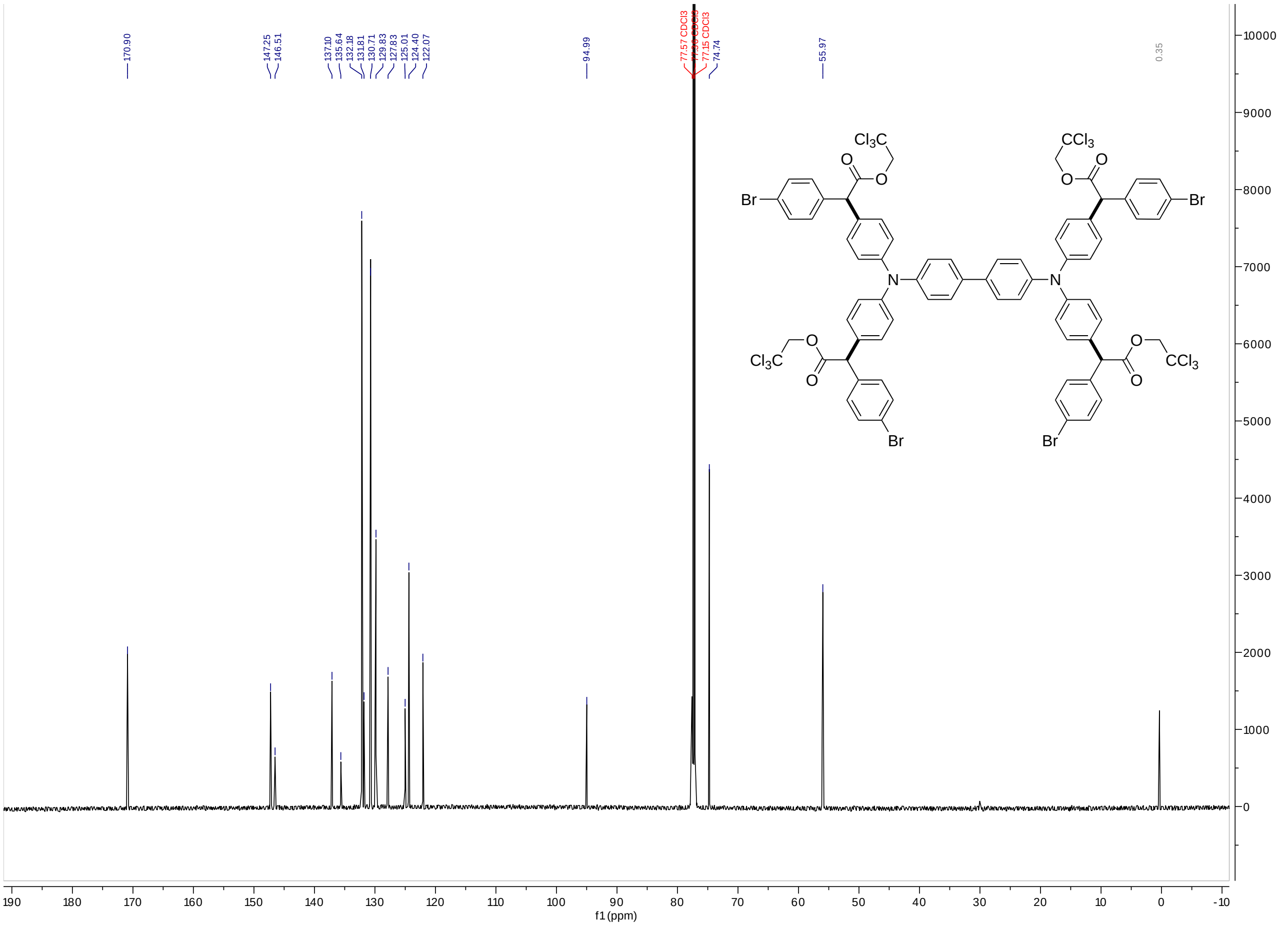

Figure S18. $^{13}C\{^{1}H\}$ NMR spectrum (151 MHz, Chloroform-*d*) of **18**.

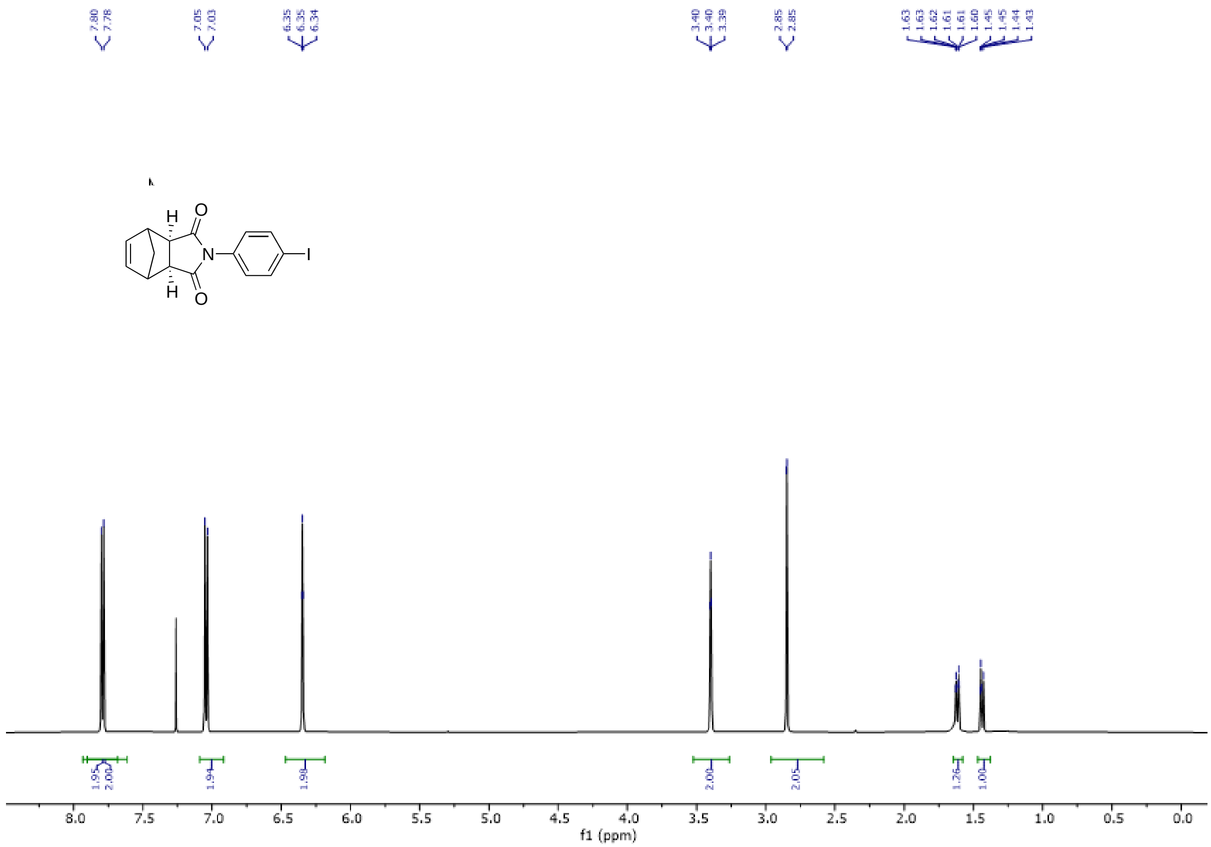

Figure S19. ^{1}H NMR spectrum (500 MHz, Chloroform-*d*) of **10**.

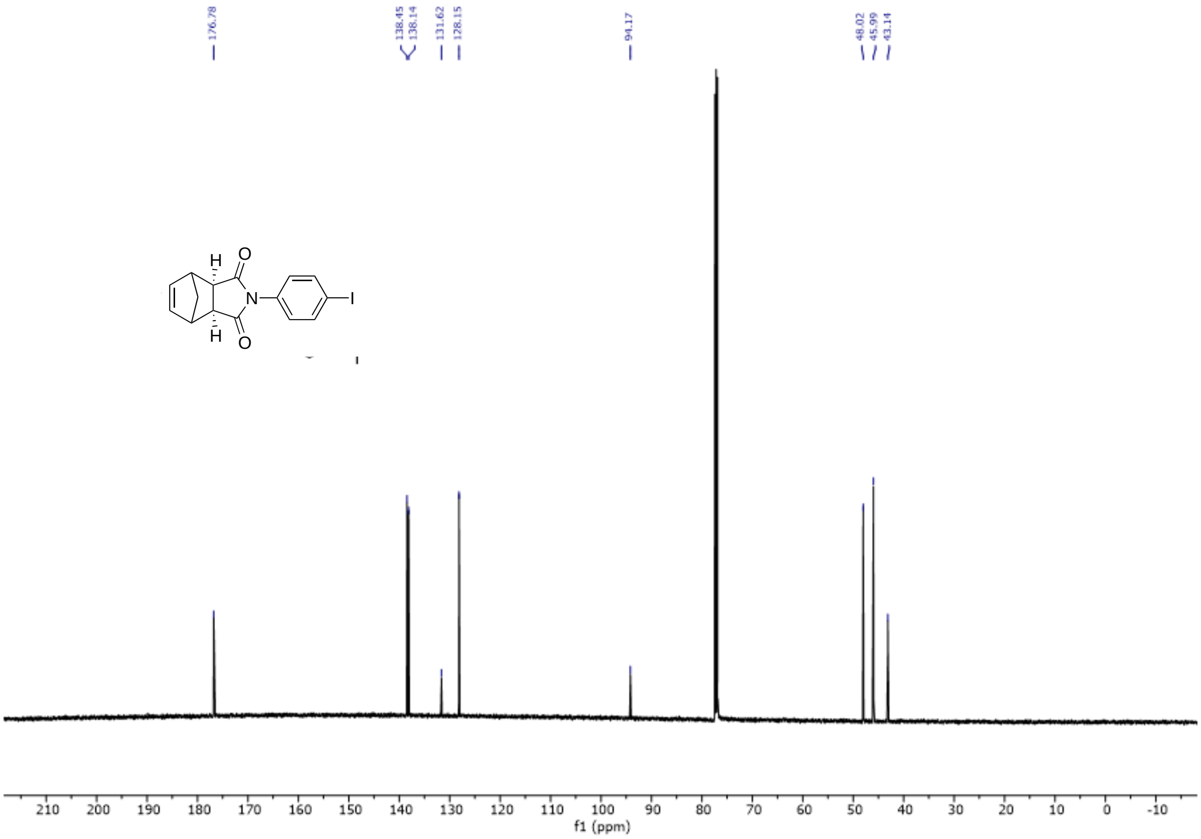

Figure 20. $^{13}C\{^{1}H\}$ NMR spectrum (126 MHz, Chloroform-*d*) of **10**.

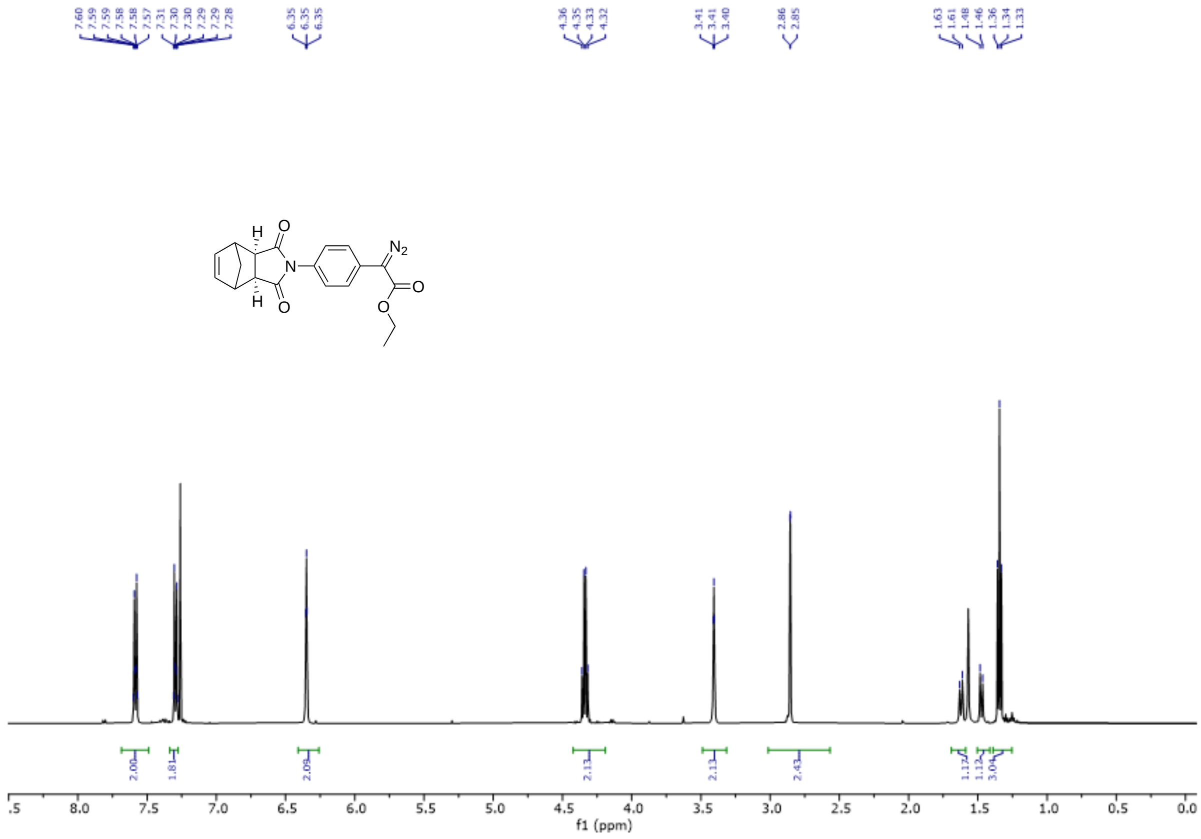

Figure 21. ^{1}H NMR spectrum (500 MHz, Chloroform-*d*) of **11**.

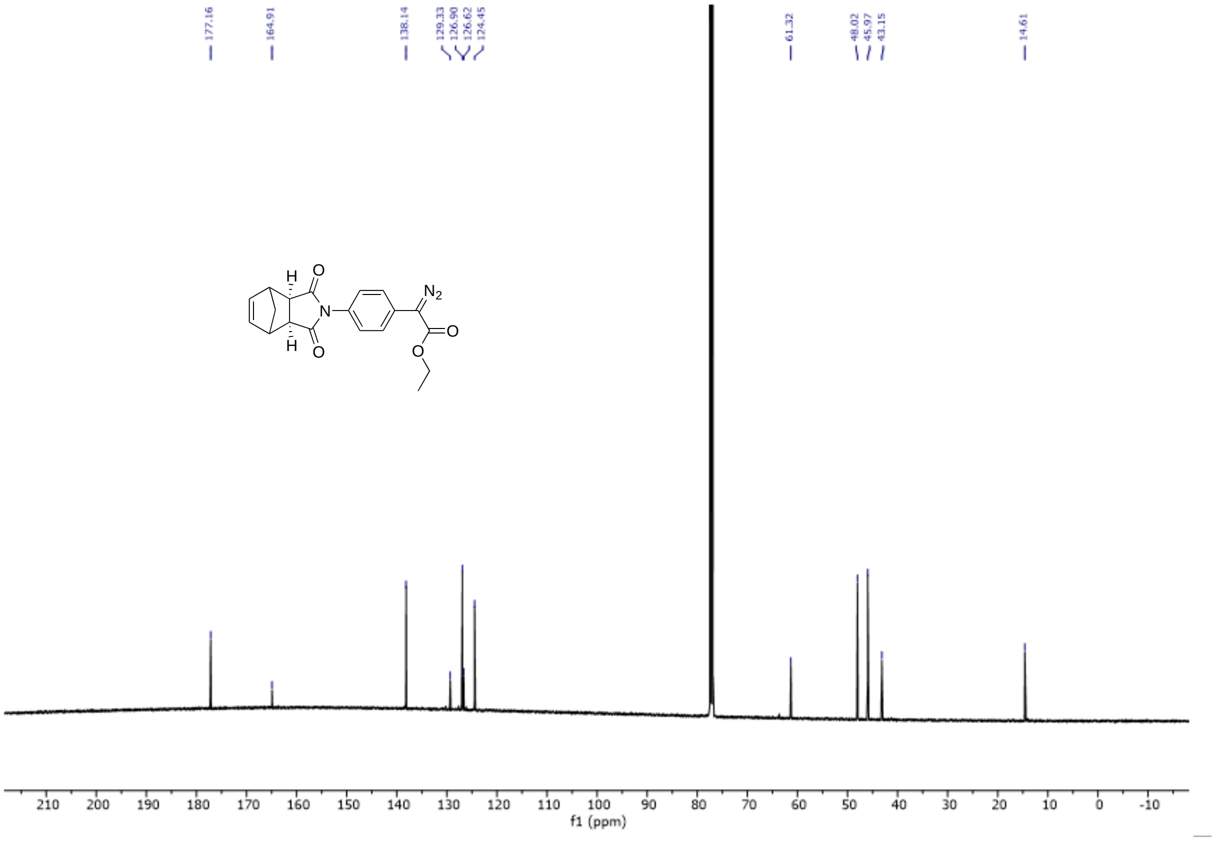

Figure 22. $^{13}C\{^{1}H\}$ NMR spectrum (126 MHz, Chloroform-*d*) of **11**.

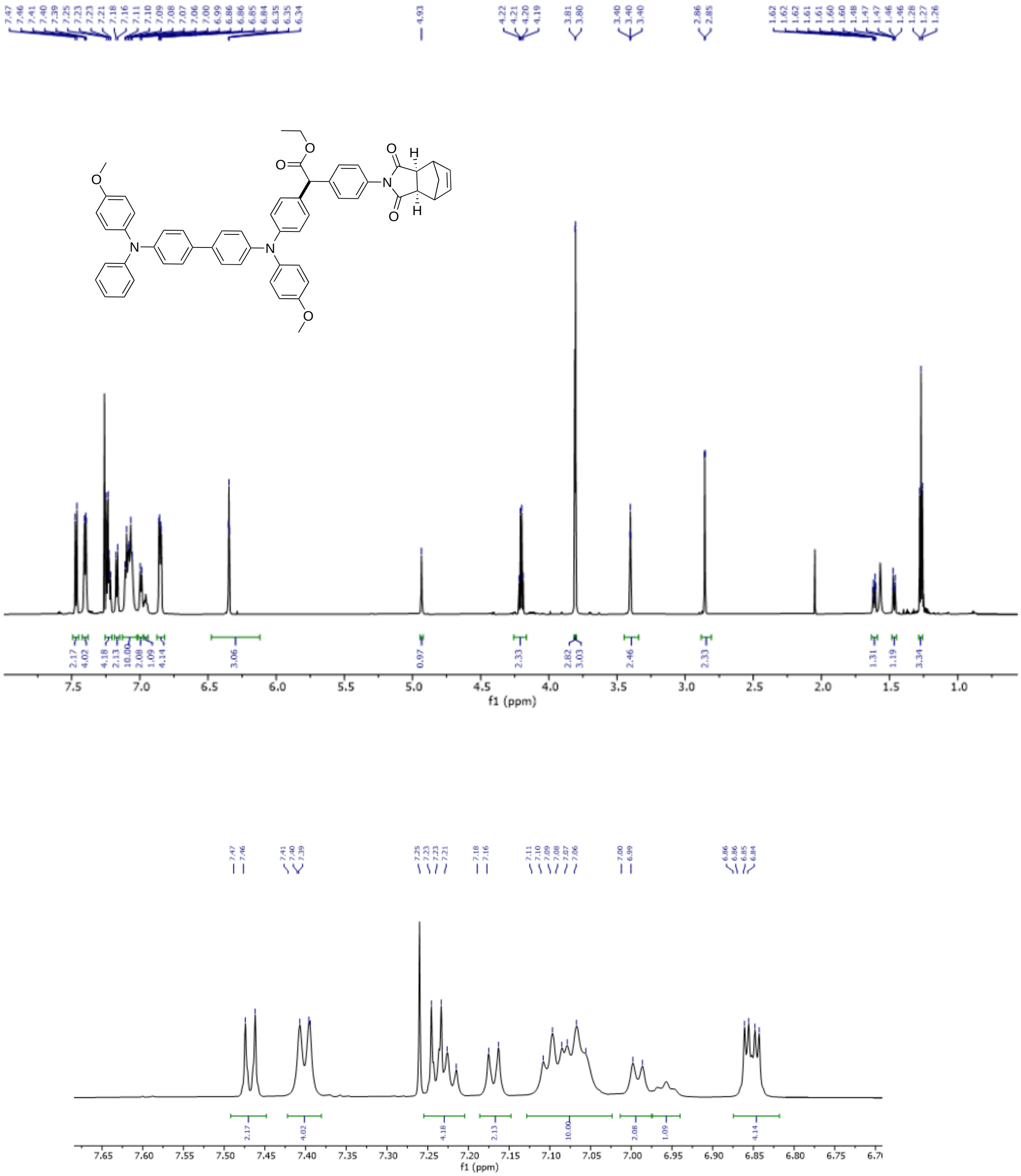

Figure 23. ^{1}H NMR spectrum (700 MHz, Chloroform-*d*) of **M1**.

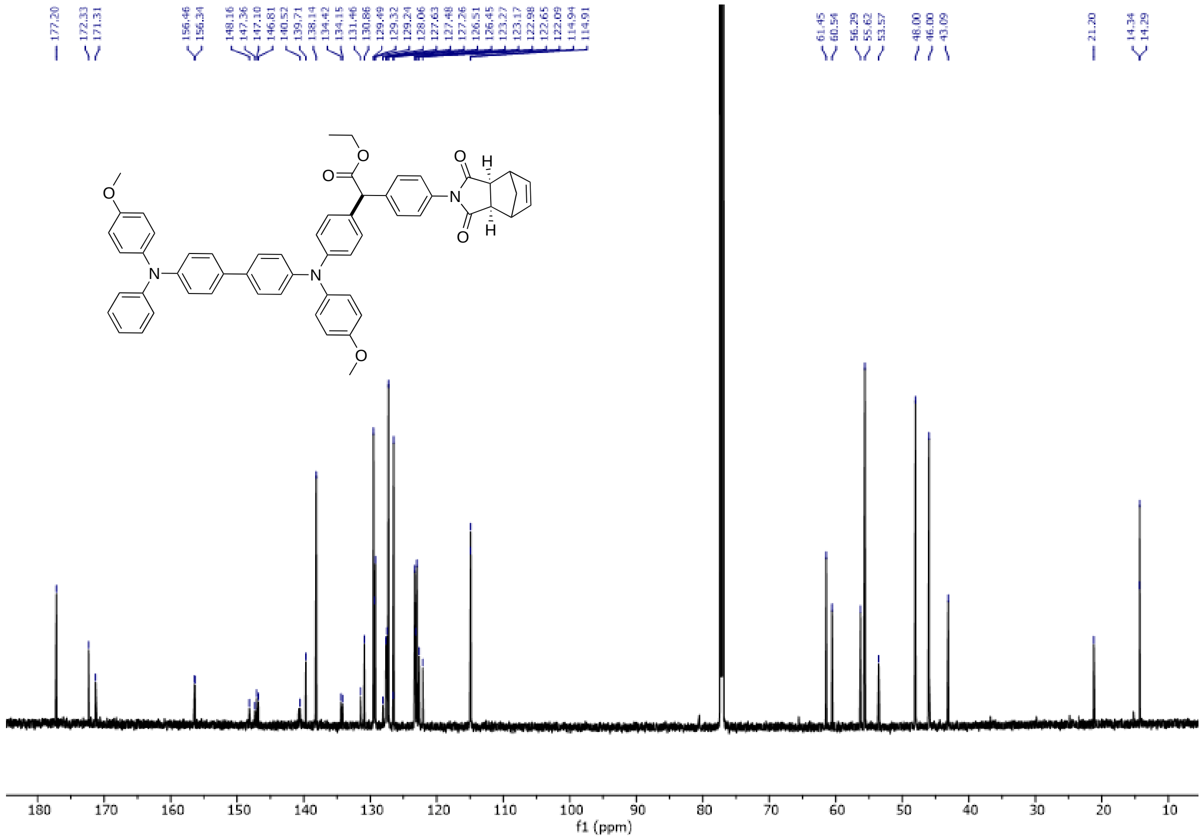

Figure 24. $^{13}C\{^{1}H\}$ NMR spectrum (176 MHz, Chloroform-*d*) of **M1**.

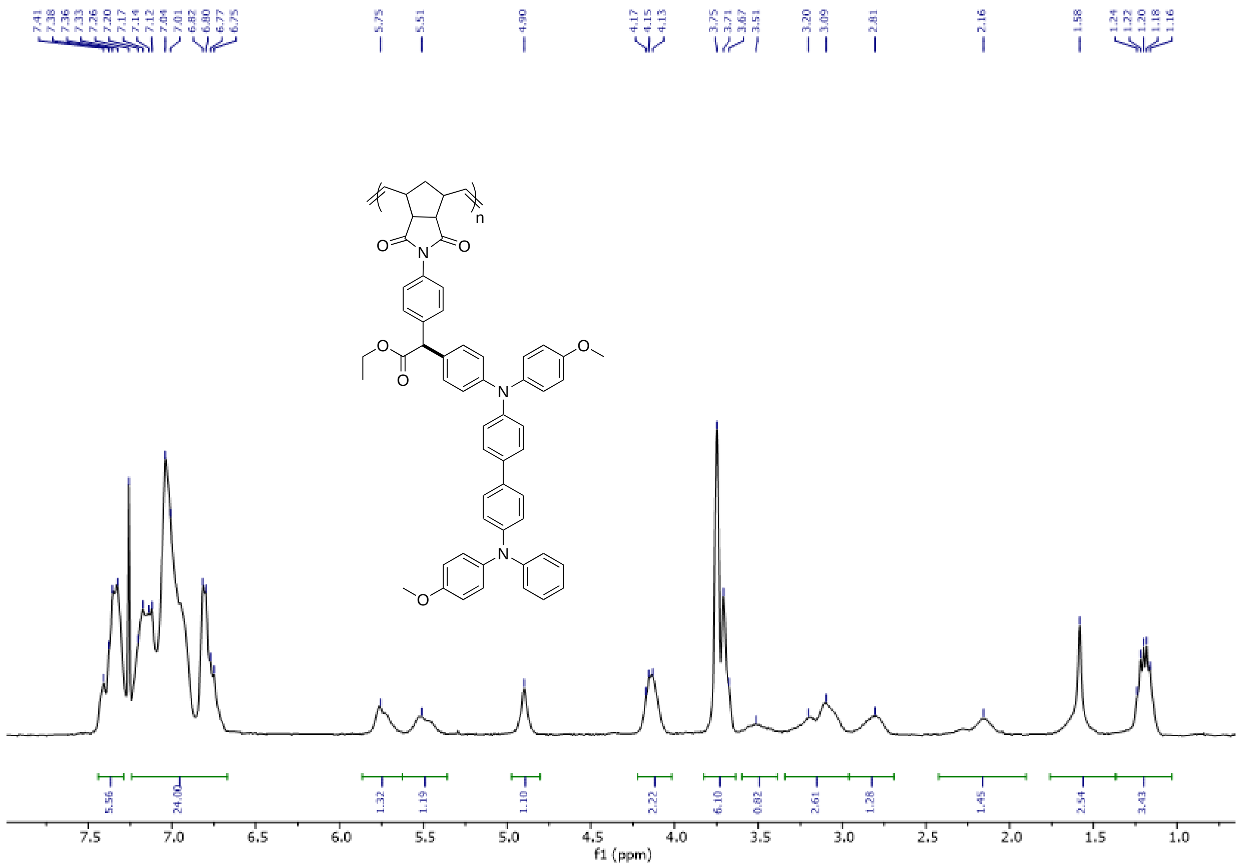

Figure 25. ^{1}H NMR spectrum (700 MHz, Chloroform-*d*) of **P1**.

SECTION 3: HPLC Traces.

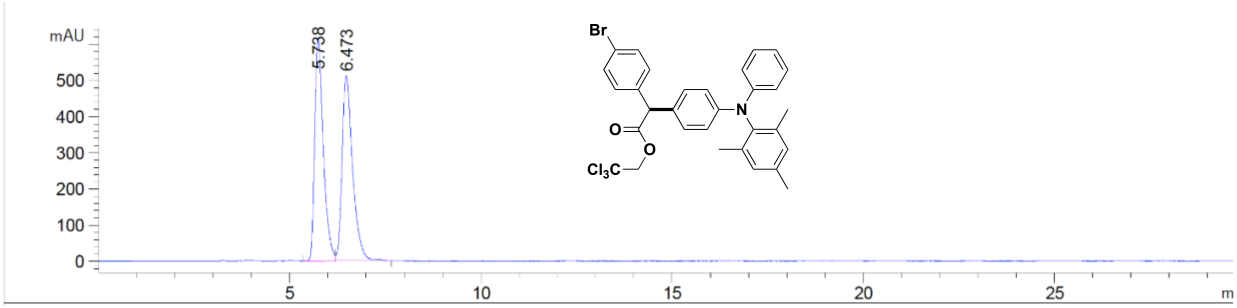

Signal 2: DAD1 B, Sig=230,4 Ref=off

Peak #	RetTime [min]	Type	Width [min]	Area [mAU*s]	Height [mAU]	Area %
1	5.738	VV R	0.2300	9639.89258	617.98462	49.4060
2	6.473	VV R	0.2640	9871.70898	511.92154	50.5940
Totals :				1.95116e4	1129.90616	

Figure 26. HPLC trace for racemic **3a**.

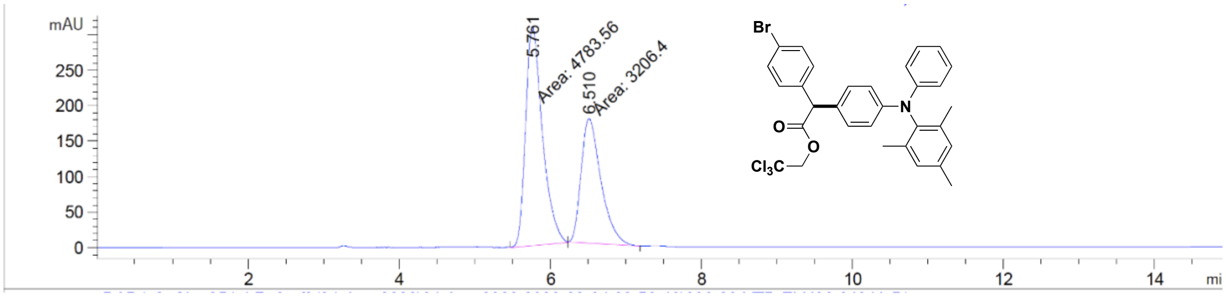

Signal 2: DAD1 B, Sig=230,4 Ref=off

Peak #	RetTime [min]	Type	Width [min]	Area [mAU*s]	Height [mAU]	Area %
1	5.761	MM	0.2580	4783.55713	309.04507	59.8696
2	6.510	MM	0.3046	3206.39819	175.44948	40.1304
Totals :				7989.95532	484.49455	

Figure 27. HPLC trace for **3a** synthesized using using Rh_2(*S*-*p*-Br-TPCP)$_4$ catalyst.
